# Genome-wide DNA methylation analysis on C-reactive protein among Ghanaians suggests molecular links to the emerging risk of cardiovascular diseases

**DOI:** 10.1038/s41525-021-00213-9

**Published:** 2021-06-11

**Authors:** Felix P. Chilunga, Peter Henneman, Andrea Venema, Karlijn A. C. Meeks, Ana Requena-Méndez, Erik Beune, Frank P. Mockenhaupt, Liam Smeeth, Silver Bahendeka, Ina Danquah, Kerstin Klipstein-Grobusch, Adebowale Adeyemo, Marcel M.A.M Mannens, Charles Agyemang

**Affiliations:** 1 Department of Public Health, Amsterdam Public Health Research Institute, Amsterdam University Medical Centers, University of Amsterdam, Amsterdam, The Netherlands; 2 Department of Clinical Genetics, Amsterdam Reproduction & Development research institute, Amsterdam University Medical Centers, University of Amsterdam, Amsterdam, The Netherlands; 3 Center for Research on Genomics and Global Health, National Human Genome Research Institute, National Institutes of Health, Bethesda, MD USA; 4 Barcelona Institute for Global Health (ISGlobal), Barcelona, Spain; 5 Department of Global Public Health, Karolinska Institutet, Solna, Sweden; 6 Institute of Tropical Medicine and International Health, Charité-Universitätsmedizin Berlin, Berlin, Germany; 7 Department of Non-communicable Disease Epidemiology, London School of Hygiene and Tropical Medicine, London, UK; 8 Department of Medicine, MKPGMS-Uganda Martyrs University, Kampala, Uganda; 9 Heidelberg Institute of Global Health (HIGH), Universitätsklinikum Heidelberg, Heidelberg, Germany; 10 Julius Global Health, Julius Center for Health Sciences and Primary Care, University Medical Center Utrecht, Utrecht University, Utrecht, The Netherlands; 11 Division of Epidemiology and Biostatistics, School of Public Health, Faculty of Health Sciences, University of the Witwatersrand, Johannesburg, South Africa

**Keywords:** Risk factors, Molecular medicine

## Abstract

Molecular mechanisms at the intersection of inflammation and cardiovascular diseases (CVD) among Africans are still unknown. We performed an epigenome-wide association study to identify loci associated with serum C-reactive protein (marker of inflammation) among Ghanaians and further assessed whether differentially methylated positions (DMPs) were linked to CVD in previous reports, or to estimated CVD risk in the same population. We used the Illumina Infinium® HumanMethylation450 BeadChip to obtain DNAm profiles of blood samples in 589 Ghanaians from the RODAM study (without acute infections, not taking anti-inflammatory medications, CRP levels < 40 mg/L). We then used linear models to identify DMPs associated with CRP concentrations. Post-hoc, we evaluated associations of identified DMPs with elevated CVD risk estimated via ASCVD risk score. We also performed subset analyses at CRP levels ≤10 mg/L and replication analyses on candidate probes. Finally, we assessed for biological relevance of our findings in public databases. We subsequently identified 14 novel DMPs associated with CRP. In post-hoc evaluations, we found that DMPs in *PC*, *BTG4* and *PADI1* showed trends of associations with estimated CVD risk, we identified a separate DMP in *MORC2* that was associated with CRP levels ≤10 mg/L, and we successfully replicated 65 (24%) of previously reported DMPs. All DMPs with gene annotations (13) were biologically linked to inflammation or CVD traits. We have identified epigenetic loci that may play a role in the intersection between inflammation and CVD among Ghanaians. Further studies among other Africans are needed to confirm our findings.

## Introduction

Cardiovascular diseases (CVD) are rapidly becoming the leading causes of morbidity and mortality among African populations^1^. Changes in life-style factors (e.g. tobacco smoking, poor diet and physical inactivity) are prominent contributors to the emerging risk of cardiovascular diseases (CVD). However, these factors do not fully account for the growing burden of CVD^2^.

Chronic inflammation is currently seen as an important factor in the pathogenesis of CVD^3,4^. The link between inflammation and CVD is complex with several molecular pathways identified^5^. One such pathway is via epigenetic modifications, which are heritable yet reversible molecular modifications to DNA, which can affect phenotypic expression without altering the DNA sequence^6^. DNA methylation (DNAm) is the most studied and best understood epigenetic modification^6^. It is affected by environment changes such as inflammation and modulates gene expression via the regulation of transcription factor binding and attraction of methyl-binding proteins that initiate chromatin compaction and gene silencing^6^. As such, studies among populations from high income countries (HIC) have identified DNAm sites that link inflammation to CVD^7^.

Although African populations may be exposed to similar triggers of inflammation as in HIC populations such as adiposity, heterogeneity in genotype and other inflammatory factors prevalent among Africans, such as intestinal parasites, chronic infections (HIV and Mycobacterium Tuberculosis), and other tropical diseases, may alter epigenetic pathways that are dissimilar to those of populations from HIC^8,9^. A small study attempted to unravel the epigenetic mechanisms of inflammation among black South African men but was underpowered to detect any differentially methylated sites^10^. As a result, little is known about epigenetic mechanisms linking chronic inflammation and CVD in African populations.

C-reactive protein (CRP) is a sensitive marker of inflammation and has been associated with increased risk of coronary heart disease, stroke and vascular mortality in population based studies^11,12^. We, therefore, performed an epigenome-wide association study (EWAS) to identify differentially methylated loci associated with CRP among Ghanaians. Subsequently, we assessed whether these detected loci were associated with the 10-year estimated risk of CVD in the same population, or with CVD in previous genome/epigenome-wide association studies^13,14^.

## Results

### Baseline characteristics

A total of 589 participants were included in the current analysis. Of these, 313 (53.1%) resided in Europe, while 276 (46.9%) resided in Ghana, which is in line with the general RODAM population. The mean age was 51 ± 18 years. The majority (55%) were female. The median CRP level was 0.80 (IQR 0.30–2.80). Those with elevated CRP (>3 mg/L) were likely to have higher BMI, to have diabetes and to have elevated 10-year predicted CVD risk and to have a lower estimated CD4 T-cell count (Table 1).

**Table 1:** Baseline characteristics of Ghanaian participants.

	Total (*n* = 589)	Low CRP category (*n* = 306)^a^	Borderline CRP category (*n* = 143)^a^	Elevated CRP category (*n* = 140)^a^	*P*-value^b^
Age, mean (SD)^c^	51.18 (9.87)	50.17 (9.40)	51.92 (10.31)	52.62 (10.21)	0.009
Sex (female), *n* (%)	324 (55.01)	146 (47.71)	81 (56.64)	97 (69.29)	<0.001
Location					0.222
Rural Ghana	76 (12.90)	34 (11.11)	20 (13.98)	22 (15.71)	
Urban Ghana	200 (33.96)	99 (32.35)	46 (32.17)	55 (39.29)	
European Ghanaians	313 (53.14)	173 (56.54)	77 (53.85)	63 (45.00)	
Education, *n* (%)					0.032
Elementary	186 (31.57)	94 (30.72)	47 (32.87)	45 (32.14)	
Primary	244 (41.42)	114 (37.25)	70 (48.95)	60 (42.86)	
Secondary	102 (17.32)	63 (20.59)	19 (13.29)	20 (14.29)	
Tertiary	57 (9.67)	35 (11.44)	7 (4.90)	15 (10.71)	
Smoking, *n* (%)					0.436
Current	15 (2.55)	5 (1.63)	6 (4.20)	4 (2.86)	
Past	58 (9.85)	34 (11.11)	13 (9.09)	11 (7.86)	
Alcohol consumption, mean (SD)^d^	0.21 (0.85)	0.21 (0.79)	0.31 (1.23)	0.09 (0.29)	0.369
Diabetes, *n* (%)	210 (35.65)	85 (27.78)	52 (36.36)	73 (52.14)	<0.001
BMI, mean (SD)^e^	27.02 (5.59)	25.42 (4.66)	27.91 (5.43)	29.62 (6.44)	<0.001
Elevated CVD risk, *n* (%)^f^	281 (47.70)	136 (44.44)	68 (47.55)	77 (55.00)	0.017
Systolic BP, mean (SD)^f^	134.97 (20.68)	133.89 (19.79)	137.17 (22.89)	135.09 (20.17)	0.399
Use of anti-hypertensives, *n* (%)^f^	68 (24.19)	41 (30.14)	12 (17.64)	15 (19.48)	0.147
Total cholesterol, mean (SD)^f^	5.23 (1.21)	5.07 (1.04)	5.37 (1.45)	5.46 (1.21)	<0.001
HDL cholesterol, mean (SD)^f^	1.34 (0.38)	1.39 (0.40)	1.32 (0.37)	1.23 (0.32)	<0.001
Cell counts in %					
CD4 T cells, mean (SD)	18.18 (5.65)	18.31 (5.48)	18.87 (5.49)	17.17 (6.07)	0.107
CD8 T cells, mean (SD)	10.73 (4.75)	10.99 (5.11)	10.34 (4.44)	10.58 (4.20)	0.013
Natural killer cells, mean (SD)	10.80 (5.50)	11.14 (5.54)	10.64 (5.50)	10.21 (5.58)	0.089
B cells, mean (SD)	10.60 (3.44)	10.67 (3.33)	10.74 (3.70)	10.29 (3.42)	0.352
Monocytes, mean (SD)	8.01 (2.42)	7.75 (2.16)	8.15 (2.77)	8.43 (2.50)	0.004
Granulocytes, mean (SD)	45.41 (9.04)	44.82 (9.16)	45.08 (8.85)	47.01 (8.81)	0.026
CRP, median (IQR)^g^	0.80 (0.30–2.80)	–	–	–	–
CRP ≤ 10 mg/L, *n* (%)	549 (93%)	–	–	–	–

^a^CRP levels categorized according to American Heart Association categories: <1 mg/L = low, 1 to 3 mg/L = borderline, >3 mg/L = Elevated.

^b^*P*-value for differences in means.

^c^Age is measured in years.

^d^Alcohol consumption in units per day.

^e^BMI = Body mass index presented in Kg/m^2^ as mean with standard deviation.

^f^10-year risk of CVD estimated using the Pooled Cohort Equations. Variables used for the estimation include age, sex, total cholesterol, high-density lipoprotein cholesterol, systolic BP, use of antihypertensive medication, diagnosed with diabetes mellitus and smoking. Low CVD risk <7.5%, Elevated CVD risk ≥7.5%. In this subset, *N* = 472. Proportion of the most abundant cells was estimated using the method by Houseman et al.^60^

^g^High sensitivity-C-reactive protein (CRP) measured in mg/L.

### Differentially methylated positions and regions

**Fig. 1:**
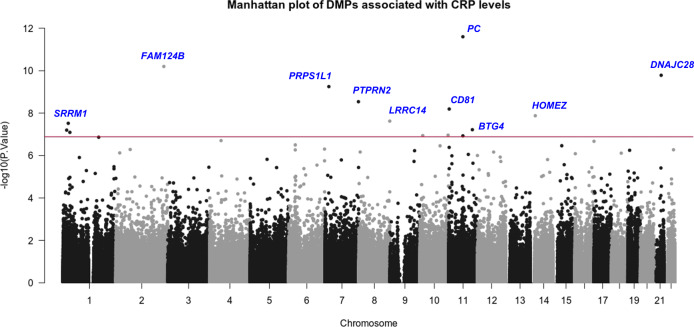
Manhattan plot of DMPs associated with CRP among Ghanaians. All 429,459 CpG sites are presented according to *p*-value in EWAS, as well as by chromosomal annotation. Red line is the demarcation line for statistically significant DMPs at *p* < 1.1E-7.

DNAm levels of 14 CpG sites showed genome-wide significant associations with CRP at 5% FDR (Table 2, Fig. 1). These DMPs included; cg14653250 in TSS200 (transcription start site) of *PC*, cg02338947 in 3′UTR (untranslated region) of *FAM124B*, cg01573121 in 5′UTR of *DNAJC28*, cg12144754 in 1stExon of *PRPS1L1*, cg26859186 in the body of *PTPRN2*, cg19712490 in TSS200 of *CD81*, cg12842013 in 1stExon of *HOMEZ*, cg01099220 in 3′UTR of *LRRC14*, cg25806492 in body of *SRRM1*, cg13767940 in TSS200 of *BTG4*, cg21010178 in TSS1500 of *PADI1*, cg22602019 in 3′UTR of *FAM167B* and cg13198133 in the intergenic region and cg02150674 in TSS200 of *PHYH* as per Illumina platform annotations (IlluminaHumanMethylation450kanno.ilmn12.hg19). DNAm variation in these DMPs ranged from 0.04% (cg02150674) to 0.4% (cg02338947) with each unit increase in CRP (mg/L), Table 2, Supplementary Fig. 1). Our statistical analyses did not return any DMRs using *bumphunter*.

**Table 2:** List of differentially methylated positions associated with CRP among Ghanaians.

No	CpG ID	Chromosome	Position^a^	Gene name^a^	Feature^a^	Relation to Island^a^	Delta β value^b^	*P*-value	FDR
1	cg14653250	chr11	66725860	*PC*	TSS200	Island	5.31E-04	2.51E-12	1.1E-06
2	cg02338947	chr2	225244102	*FAM124B*	3′UTR	OpenSea	−4.47E-03	6.36E-11	1.4E-05
3	cg01573121	chr21	34863117	*DNAJC28*	5′UTR	N_Shore	−1.32E-03	1.64E-10	2.4E-05
4	cg12144754	chr7	18067437	*PRPS1L1*	1stExon	OpenSea	−9.53E-04	5.63E-10	6.0E-05
5	cg26859186	chr7	157351708	*PTPRN2*	Body	N_Shore	−1.61E-03	2.91E-09	2.5E-04
6	cg19712490	chr11	2398477	*CD81*	TSS200	Island	2.32E-03	6.41E-09	4.6E-04
7	cg12842013	chr14	23755227	*HOMEZ*	1stExon	Island	4.33E-04	1.33E-08	8.1E-04
8	cg01099220	chr8	145747659	*LRRC14*	3′UTR	Island	1.26E-03	2.38E-08	1.3E-03
9	cg25806492	chr1	24970113	*SRRM1*	Body	Island	1.57E-03	3.03E-08	1.4E-03
10	cg13767940	chr11	111383603	*BTG4*	TSS200	Island	2.15E-03	6.06E-08	2.5E-03
11	cg21010178	chr1	17530227	*PADI1*	TSS1500	OpenSea	−1.93E-03	6.35E-08	2.5E-03
12	cg22602019	chr1	32714308	*FAM167B*	3′UTR	S_Shore	9.00E-04	8.12E-08	2.9E-03
13	cg13198133	chr10	132453765	Intergenic	Intergenic	OpenSea	−2.92E-03	1.10E-07	3.4E-03
14	cg02150674	chr10	13342205	*PHYH*	TSS200	Island	4.94E-04	1.15E-07	3.4E-03

*TSS1500* transcription start site 1500 (the region from Transcription start site (TSS) to −1500 nucleotides upstream of TSS), *5*′*UTR* 5′ untranslated region (the region of an mRNA that is directly upstream from the initiation codon), *3*′*UTR* 3′ untranslated region (the region of an mRNA that is downstream from the stop codon), *TSS200* transcription start site 200 (the region from Transcription start site (TSS) to −200 nucleotides upstream of TSS), *FDR* false discovery rate (a 5% FDR is considered significant).

^a^Annotation were perfomed via IlluminaHumanMethylation450kanno.ilmn12.hg19. *Homo sapiens* (human) genome assembly GRCh37 (hg19)^61^.

^b^Delta β-value of DNA methylation against each unit increase in CRP (mg/L) derived from linear regression models adjusted for age, sex, array, plate position, alcohol consumption, smoking, BMI, type 2 diabetes and proportion of immune cells (N = 589).

### Post-hoc findings

First, we performed a replication analysis on 280 DMPs that were identified in a multi-ethnic EWAS meta-analysis on CRP on populations from HIC (Supplementary Table 1). While eight out of these 280 candidates could not be evaluated in our data (removed during quality control), the rest (272) were not part of our top 14 genome-wide significant DMPs. Performing linear regression on these 272 candidates, we identified 66 candidate probes with *p* < 0.05. Except for one probe (cg22959742), the rest of the probes (65) had similar direction of effects as in the multi-ethnic EWAS meta-analysis on CRP. Thus, we replicated 24% (65/272) of the DMPs based on consistency of direction of effect (Supplementary Table 2).

Second, we performed sensitivity analyses on obesity and type 2 diabetes (T2D; Supplementary Fig. 2). We found that our top 14 genome-wide significant DMPs were also detectable in sensitivity analyses that excluded BMI and T2D as covariates in linear regression models. In addition, we found strong correlations between delta-beta values (r = 0.99), and between *p*-values (r = 0.81) in the main analysis in relation to the sensitivity analyses. Moreover, none of the top 14 genome-wide significant DMPs was reported in previous EWAS on obesity and T2D in the same study population^15,16^.

Third, we performed sensitivity analyses on location of residence (Supplementary Table 3 and Supplementary Fig. 3). With regards to our top 14 genome-wide significant DMPs, we detected some slight differences in delta-beta values between Ghanaians resident in Ghana and Ghanaians resident in Europe. For instance, the delta-beta values for cg14653250 (*PC*), cg01573121 (*DNAJC28*), cg12842013 (*HOMEZ*), cg25806492 (*SRRM1*), cg13767940 (*BTG4*) and cg21010178 (*PADI1*) were slightly higher in Ghanaians resident in Europe as compared to Ghanaians resident in Ghana, while those for cg12144754 (*PRPS1L1*), cg26859186 (*PTPRN2*), cg19712490 (*CD81*), cg22602019 (*FAM167B*), cg13198133 (intergenic) and cg02150674 (*PHYH*) were slightly higher in Ghanaians resident in Ghana compared to Ghanaians resident in Europe. We did not detect any differences in delta-beta values in cg02338947 (*FAM124B*) and cg01099220 (*LRRC14*) between Ghanaians resident on the two continents. However, all 14 genome-wide significant DMPs were still detectable in sensitivity analyses that included migration status as a covariate in linear regression models. Characteristically, delta-beta values in the main analysis were strongly correlated to those in sensitivity analysis (r = 0.99). Such strong correlations were also observed for *p*-values (r = 0.99).

Fourth, we examined DNAm differences at CRP levels ≤10 mg/L (Supplementary Figs. 4, 5, Supplementary Table 4). This was achieved by performing a subset analyses on 549 participants with CRP levels ≤10 mg/L. DNAm levels at one CpG site (cg02551882 in TSS1500 of *MORC2* gene) showed genome-wide significant associations with CRP concentrations ≤10 mg/L at 5% FDR. It had a delta-beta value of 0.04% for each unit increase in CRP concentration (mg/L).

Fifth, we investigated the link between DNAm variations in CRP and estimated cardiovascular risk (Table 3). This was achieved by assessing whether DNAm in genome-wide significant DMPs was associated with estimated CVD risk in a sub-sample of participants with ASCVD risk scores (*n* = 472 eligible based on ASCVD criteria) at 5% FDR. Although three probes (cg14653250 in *PC*, cg13767940 in *BTG4*, cg21010178 in *PADI1*) showed trends of associations with estimated CVD risk (FDR 0.07–0.08), none of the genome-wide significant DMPs demonstrated statistical significance at an FDR threshold of <0.05 in these particular analyses.

**Table 3:** Associations between DNA methylation in genome-wide significant DMPs and estimated risk of cardiovascular diseases (ASCVD) among Ghanaians.

CpG ID	Nearest gene^a^	Gene feature^a^	Beta coefficient^b^	*P*-value	FDR
cg14653250	*PC*	TSS200	0.0028	0.005	0.071
cg02338947	*FAM124B*	3′UTR	0.0023	0.726	0.848
cg01573121	*DNAJC28*	5′UTR	−0.0004	0.872	0.920
cg12144754	*PRPS1L1*	1stExon	0.0019	0.294	0.515
cg26859186	*PTPRN2*	Body	0.0016	0.464	0.650
cg19712490	*CD81*	TSS200	−0.0059	0.209	0.421
cg12842013	*HOMEZ*	1stExon	0.0009	0.205	0.421
cg01099220	*LRRC14*	3′UTR	−0.0003	0.919	0.920
cg25806492	*SRRM1*	Body	0.0034	0.210	0.421
cg13767940	*BTG4*	TSS200	0.0098	0.016	0.084
cg21010178	*PADI1*	TSS1500	−0.0089	0.018	0.084
cg22602019	*FAM167B*	3′UTR	−0.0023	0.208	0.421
cg13198133	Intergenic	Intergenic	0.0017	0.698	0.848
cg02150674	*PHYH*	TSS200	−0.0009	0.369	0.575

*ASCVD risk* American College of Cardiology/American Heart Association atherosclerotic cardiovascular disease (ACC/AHA ASCVD) risk score as previously applied in the RODAM study. The risk score is used among persons aged 40–79 years, without prior history of CVD, using an algorithm that combines age, sex, use of antihypertensive medication, systolic blood pressure, presence of T2D, total cholesterol, HDL cholesterol and smoking status. A score of >7.5% is considered to be an elevated risk of developing a CVD in the next 10 years based on the prior work by Goff et al.^59^.

^a^Annotation were perfomed via IlluminaHumanMethylation450kanno.ilmn12.hg19. *Homo sapiens* (human) genome assembly GRCh37 (hg19)^61^.

^b^β-coefficient from linear regression model of DNA methylation in CRP (outcome) against estimated risk of cardiovascular diseases adjusted for alcohol consumption, BMI and proportion of immune cells. *N* = 472 (281 for elevated CVD risk, 191 for low CVD risk).

Sixth, we investigated biological relevance of our findings with respect to gene expression, and links to inflammation and CVD traits in public databases (Table 4, Supplementary Tables 5–8). We first evaluated the link between genome-wide significant DMPs and gene expression in IMETHYL database. We found that lower DNAm in 5′UTR of *DNAJC28* (cg01573121), and in TSS of *PC* (cg14653250), *PHYH* (cg02150674) and *MORC2* (cg02551882) were associated with higher expression of the respective genes, while higher DNAm in the body *PTPRN2* (cg26859186) and *HOMEZ* (cg12842013), and in 3′UTR of *FAM167B* (cg22602019) were associated with higher expression of respective genes. This pattern was not observed in the remaining DMPs. Next, we assessed whether genes annotated to statistically significant DMPs were enriched to discrete pathways in KEGG and GO databases using *MissMethyl* package at 5% FDR. Both KEGG and GO databases did not yield any statistically significant pathways. However, the pathways that were observed at higher FDR (>5%) included adaptive immune response, CD4-positivity, alpha-beta T-cell stimulation, methyl-branched fatty acid metabolic process, fatty acid biosynthesis, B-cell receptor signaling pathway, Hepatitis C and metabolic pathways which seem biologically plausible in the context of our study since these pathways are related to chronic inflammation. Finally, we searched in genome -wide association studies (GWAS) catalog (https://www.ebi.ac.uk/gwas/), GeneCards (https://www.genecards.org/) and EWAS catalog (http://ewascatalog.org/) to determine whether variants in genes annotated to statistically significant DMPs were linked to inflammation or CVD traits. We found that all genome-wide significant DMPs with gene annotations (13) were linked to inflammation or CVD factors such as markers of inflammation (interferons, interleukins, platelet-derived growth factors, leukocyte count, fibrinogen), chronic inflammatory diseases (HIV infections, allergies/asthma, autoimmune diseases) and cardiometabolic factors (cholesterol levels, Apo lipoproteins, blood pressure, carotid plaque build, coronary artery calcification, N-terminal pro-B-type natriuretic peptide and incident CVD).

**Table 4:** Relationship between DNA methylation and gene expression as reported in the IMETHYL database.

CpG ID	Nearest gene^a^	Gene feature^a^	Methylation level ^b^	Methylation average^b^% (SD)	FPKM^b,c^ average(SD)
cg14653250	*PC*	TSS200	Low	0.77 (1.51)	0.61 (0.18)
cg02338947	*FAM124B*	3′UTR	High	84.10 (24.48)	−0.42 (0.18)
cg01573121	*DNAJC28*	5′UTR	High	86.66 (8.10)	−0.59 (0.15)
cg12144754	*PRPS1L1*	1stExon	High	97.81 (2.43)	Data not available
cg26859186	*PTPRN2*	Body	High	96.10 (3.50)	0.27 (0.17)
cg19712490	*CD81*	TSS200	Low	0.43 (1.48)	−0.61 (0.17)
cg12842013	*HOMEZ*	1stExon	Low	0.39 (1.22)	0.80 (0.09)
cg01099220	*LRRC14*	3′UTR	Low	2.09 (2.70)	1.25 (0.07)
cg25806492	*SRRM1*	Body	Low	2.55 (2.28)	1.87 (0.07)
cg13767940	*BTG4*	TSS200	Low	6.40 (4.93)	Data not available
cg21010178	*PADI1*	TSS1500	High	94.17 (5.16)	Data not avaialable
cg22602019	*FAM167B*	3′UTR	Low	13.63 (0.42)	−0.26 (0.17)
cg13198133	Intergenic	Intergenic	High	91.00 (5.71)	Data not vailable
cg02150674	*PHYH*	TSS200	Low	0.71 (1.92)	0.73 (0.10)

^a^Annotation were perfomed via IlluminaHumanMethylation450kanno.ilmn12.hg19. *Homo sapiens* (human) genome assembly GRCh37 (hg19)^61^.

^b^Methylation level according to iMETHYL database (low, medium, high). IMETHYL provides whole-DNA methylation (~24 million autosomal CpG sites), whole-genome (~9 million single-nucleotide variants) and whole-transcriptome (>14 000 genes) data for CD4^+^ T-lymphocytes, monocytes and neutrophils collected from ~100 subjects.

^c^*FPKM* Fragments Per Kilobase of transcript per Million mapped reads.

## Discussion

We identified 14 novel DMPs associated with CRP levels up to 40 mg/L in Ghanaians without acute infections or taking anti-inflammatory medications. In post-hoc evaluations, we found that DMPs in *PC, BTG4* and *PADI1* showed trends of associations with estimated CVD risk, we identified a separate DMP in *MORC2* that was associated with CRP levels ≤10 mg/L, and we successfully replicated 65 (24%) of previously reported CRP associated DMPs. All DMPs with gene annotations (13) were biologically linked to inflammation or CVD traits.

In our study of DNAm variations associated with CRP in Ghanaians without acute infections or taking anti-inflammatory medications, we had postulated that epigenetic pathways may be dissimilar to those of populations from HIC. This would potentially be due to heterogeneity in genotype between Africans and population from HIC^17^, as well as to differences in inflammatory factors that may be prevalent in each population. For example, intestinal parasites, chronic infections (HIV, Hepatitis C and Mycobacterium Tuberculosis) and other tropical diseases may be common among Africans than in populations from HIC^8,9^. Moreover, chronic infections that have higher prevalence in Africans than in populations from HIC (e.g. HIV, Hepatitis C, etc.) are known to raise CRP concentration to >10 mg/L^18^, which was the basis for inclusion of CRP levels up to 40 mg/L in our study (acute bacterial infections are mostly the cause after this threshold)^18^. This is in contrast to populations from HIC who might have less of these infections^19^.

Subsequently, we identified 14 novel DMPs that were associated with CRP concentrations up to 40 mg/L in Ghanaians without acute infections or taking anti-inflammatory medications. Of these DMPs, 13 were annotated to genes, such as *PC*, *FAM124B*, *DNAJC28*, *PRPS1L1, PTPRN2, CD81, HOMEZ*, *LRRC14*, *SRRM1, BTG4*, *PADI1, FAM167B* and *PHYH*. DNAm in these 13 genes was generally in line with gene expression trends reported in literature, whereby higher DNAm in the promoter region was associated with lower gene expression and higher DNAm in the gene body was associated with higher gene expression^20^. While these genes have various functions in the human body, the genes *CD81* and *LRRC14* have specific inflammation related functions. Specifically, the gene *CD81* encodes a protein that plays a role in the regulation of cell development and motility in interleukin 2 (IL-2) pathway^21^. IL-2 has essential roles in key functions of the immune system, primarily via its direct effects on T cells^22^. On the other hand, *LRRC14* gene encodes a leucine-rich repeat-containing protein, which negatively regulates a toll-like receptor-mediated nuclear factor-kappa-B (NF-κB) signaling^21^. NF-κB is a major transcription factor that regulates genes responsible for both the innate and adaptive immune response^23^. Despite only two genes (of the 13) having immune related functions, previous EWAS and GWAS have shown that all 13 genes are linked to inflammatory traits. For instance, genomic/epigenomic variation in *PC* and *SRRM1* was associated with HIV infection and rheumatoid arthritis^24,25^, in *FAM124B* with monokine induced by gamma interferon^26^, in *DNAJC28* with ulcerative colitis and crohn’s disease^27^, in *PRPS1L1* with fibrinogen levels^28^, in *PTPRN2* with atopic eczema and inflammatory bowel disease^25,29^, in *CD81* with systemic scleroderma and monocyte count^30,31^, in *HOMEZ* with HIV infection^24^, in *LRRC14* with neutrophils and eosinophil counts^30^, in *BTG4* with IL-4 levels^26^, in *PADI1* with serum 25-Hydroxyvitamin D levels^32^, in *FAM167B* with rheumatoid arthritis^25^ and in *PHYH* with allergies^33^. It is therefore possible that DNAm variations in these genes plays a role in chronic inflammation among Ghanaians. On the other hand, a single DMP (cg13198133) was intergenic with no deoxyribonuclease (DNase) sensitivity markers in its vicinity (i.e. no evidence of links to gene expression in *cis*) and has not been reported in previous EWAS. It is not clear whether this probe is biologically meaningful with respect to chronic inflammation.

The other goal of our study was to ascertain the link between DNAm variations in CRP and CVD among Ghanaians. Although all 14 genome-wide significant DMPs did not reach the statistical criteria for detecting associations between DNAm and estimated CVD risk (FDR < 0.05), DNAm variation in probes annotated to *PC*, *BTG4* and *PADI1* genes showed some trends of association (FDR 0.07–0.08). While *BTG4* and *PADI1* do not have CVD related functions, the *PC* gene encodes pyruvate carboxylase, an important enzyme in gluconeogenesis, lipogenesis and insulin secretion^34^. Deregulation of *PC* expression has been associated with T2D, which is a major risk factor for CVD and an important component of the CVD risk prediction algorithm^35^. Furthermore, previous GWAS and EWAS have shown that variants in all three genes are linked to CVD risk factors. For example, variants in *PC* have been associated with proinsulin levels, urate levels and waist-to-hip ratio^36,37^, in *BTG4* with waist-to-hip ratio and IL-4 (pro-inflammatory on vascular endothelium and may play a critical role in the development of atherosclerosis)^26,37^, and in *PADI1* with dietary patterns^38^. It is therefore possible that DNAm variations in these three genes may also contribute to CVD risk in Ghanaians. Additionally, some DMPs, which did not show associations with estimated CVD risk in Ghanaians, have been previously linked to CVD. For instance, variants in *FAM124B, PTPRN2, SRRM1* have been associated with platelet-derived growth factors (atherosclerotic pathways)^26^, carotid plaque buildup and strokes^39,40^ and incident CVD, respectively, in previous EWAS and GWAS^41^. Since our analysis was based on estimated CVD risk (which might vary from actual incident CVD after 10 years), future studies should assess whether DNAm in these genes could also affect incident CVD in Ghanaians.

Due to environmental differences in Europe and Ghana, which might uniquely affect CRP levels in each context^9^, we assessed whether location of residence in Ghana or in Europe among our Ghanaians participants influenced our findings. While we found very tiny differences in effect sizes (delta-beta values) between Ghanaians resident in Ghana and Ghanaians resident in Europe in several DMPs (i.e. those annotated to *PC, DNAJC28, PRPS1L1, PTPRN2, CD81, HOMEZ, SRRM1, BTG4, PADI1* and FAM*167B* genes), the effects were still apparent and unchanged after adjusting for location of residence in the main analyses. This shows that location of residence did not exert any influence on our findings.

Since our study had included CRP concentrations up 40 mg/L (mostly seen in persons with chronic diseases as regards to chronic inflammation)^42^, we performed a subset analysis at CRP levels ≤10 mg/L (mostly seen in healthy adults and represent metabolic inflammation)^43^. We identified cg02551882 in the promoter region of *MORC2* to be associated with CRP levels ≤10 mg/L, with a delta-beta value of 0.04% for each unit increase in CRP (mg/L). Higher DNAm in cg02551882 was associated with lower gene expression in *MORC2* per IMETHYL database. The *MORC2* gene encodes a protein that regulates condensation of heterochromatin in response to DNA damage and plays a role in transcription repression^21^. This protein also plays a role in lipogenesis and adipocyte differentiation^44^. Moreover, previous EWAS and GWAS have shown that variants in *MORC2* are associated with metabolic inflammatory traits such as BMI, T2D and coronary artery calcification^45,46^, as well as non-metabolic inflammatory traits such as eosinophil count and primary Sjogren’s syndrome^41,47^. Our findings therefore show that DNAm methylation aberrations in CRP among Ghanaians are more apparent at chronically elevated CRP levels beyond 10 mg/L (14 DMPs) than below this threshold (one DMP). Further studies should characterize the specific conditions that lead to CRP levels beyond 10 mg/L in Ghanaians^48^, which should then in turn be routinely screened and treated to prevent CVD sequelae from DNAm aberrations.

We discovered that the effects sizes (delta-beta values) were relatively small for the statistically significant DMPs. This is not surprising considering that the effect sizes in the previous EWAS meta-analysis of CRP (N > 12,000) were in the same range (0.0011–0.01)^7^. Moreover, such small DNAm differences in the EWAS meta-analysis were correlated with gene expression in *cis*^7^. This suggests that our findings may be biologically meaningful with respect to inflammation or CVD pathogenesis.

We were able to replicate 24% of previously reported DMPs from European and African American populations. This demonstrates that some DNAm aberrations associated with CRP concentrations are similar across populations. However, our findings also suggest that majority of DNAm alterations in CRP vary according to ethnicity (genetic heterogeneity), or due to environmental context (prevalent inflammatory triggers). More importantly, the role of ethnicity and context was corroborated by the small study among black south African men, which reported several distinctions in EWAS among ethnicities (in different environmental contexts) for similar traits^10^.

The main strength of this study is that it was conducted in under-represented cohorts such as populations originating from low- and middle-income countries (LMIC). This study, therefore, adds to the much-needed evidence on DNAm and inflammation in African populations. Our study has several limitations. First, DNAm was measured in blood although the preferable tissue for CRP is the liver where it is synthesized. Nevertheless, blood has been demonstrated to be a good surrogate tissue^49^. Second, the study sample of DNAm was selected based on obesity and T2D case-control status, which can influence CRP levels. However, our sensitivity analyses demonstrated that BMI and T2D did not have overt influence on our results. Third, we excluded all participants who could possibly have had acute infections, or were taking anti-inflammatory medications, or had CRP levels >40 mg/L, but we cannot exclude that some participants were still missed. Fourth, genome-wide gene expression data, which could have enhanced biological interpretation of our results is not available for this study population. Nonetheless, we utilized the validated IMETHYL database relating our top probes to gene expression^50^. This publicly available database does suggest a link between some of our loci and gene expression. Fourth, although we removed probes that hybridized to known SNPs, data on SNPs from Africans is limited and some of our results could be due to uncharacterized SNPs in Africans. However, our data did not show any clustering patterns, which are specific to SNPs^51^. Fifth, post-hoc analyses that tested associations between genome-wide significant DMPs and estimated CVD risk were performed in a subset of participants (*n* = 472) and not the total sample in which the DMPs were identified (*n* = 589), it is therefore possible that there was selection bias as DNAm variations associated with CRP were detected in the total sample. However, those selected for the post-hoc analyses met the criteria for ASCVD and were more likely to have CVD in the next 10 years as opposed to those that did not meet the criteria. Lastly, we cannot fully rule-out residual and unmeasured confounding due to the cross-sectional nature of our study design.

Our study provides the first insights into the epigenetic mechanisms linking chronic inflammation and CVD risk in Ghanaians. These findings are highly relevant because they inform our understanding of the etiology of the emerging CVD among Ghanaians with respect to chronic inflammation. Future studies are needed to confirm our findings in other African populations, as well as to elucidate specific causes of chronically elevated CRP among Ghanaians, which will facilitate screening and treatment.

## Methods

### Study population and sample selection

The study is part of the Research on Obesity and Diabetes among African Migrants (RODAM) study. The cross-sectional multi-centre RODAM study was initiated in 2012 with the aim of understanding the complex interplay between the environment and genetics in the development of obesity and diabetes among African migrants. The full details of the study have been published elsewhere^52^. In brief, the RODAM study enrolled 6385 migrant Ghanaian men and women residing in Europe and Ghana. In Europe, participants were recruited from the cities of Amsterdam (NL), Berlin (DE) and London (UK). In Ghana, recruitment of participants in the urban area was conducted in two purposively chosen cities (Kumasi and Obuasi), while recruitment in the rural area was conducted in 15 villages in the Ashanti region. Participants were included in the present analyses if they were aged ≥25 years, had completed the questionnaire, were physically examined and had blood samples taken. For the present study, we used a subset of the RODAM study for which DNAm data were available (*n* = 736). In the primary study, the 736 participants were selected for DNA profiling based on a case-control design (~300 diabetic cases, ~ 300 controls, ~135 obese controls)^15^.

From the 736 participants with DNAm data that were available for this study, 593 participants remained after quality control and excluding participants with possible acute infections (fever, upper respiratory tract symptoms, wound care, any illness in the last 2 weeks), and those taking medications, which may alter CRP levels (immune-modulating agents, NSAIDS, steroids and statins (potent anti-inflammatory agents)^53^, Supplementary Fig. 6). While CRP concentrations between 2 and 10 mg/L are considered as metabolic inflammation (i.e. metabolic pathways that cause arteriosclerosis)^43^, chronically elevated CRP concentrations >10 mg/L have also been associated with CVD^42^. This is particularly important in our study because chronic infections that have higher prevalence in Africans than in populations from HIC (e.g. HIV and Hepatitis C, etc) are also known to raise CRP concentration to >10 mg/L^18^. As such, we sought to include participants with CRP concentrations >10 mg/L in our analyses while limiting these concentrations to 40 mg/L as CRP levels above this threshold are most likely to be from acute bacterial infections in low- and middle-income countries (LMIC)^18^.

Additional removal of participants with CRP levels >40 mg/L (which were also conspicuous outliers in the CRP distribution, Supplementary Fig. 7) led to a final sample of 589 used in the current analyses (Supplementary Fig. 6).

### Ethical approval and consent to participate

Ethical approval was obtained from ethics committees of involved institutions in Ghana (Kwame Nkrumah University of Science & Technology: CHRPE/AP/200/12), Netherlands (Amsterdam University Medical Center: W12-062#12.17.0086), Germany (Charité University Berlin: EA1/307/12) and UK (London School of Hygiene & Tropical Medicine: 6208) before the start of data collection. All participants gave written informed consent.

### Phenotypic measurements

A standardised approach for questionnaires, anthropometric measurements and venepuncture samples was used across all study sites. The following measurements were obtained through a structured questionnaire; age, sex, location of residence, educational attainment, use of antihypertensive medication, previously diagnosed diabetes mellitus, alcohol consumption and smoking. Education was categorised as follows; (1) none or elementary, (2) primary, (3) secondary and (4) tertiary. Alcohol consumption was calculated in units/day. Smoking was categorised into current smokers, past smokers, or non-smokers. Body mass index (BMI) was calculated as weight (kg) divided by height in meters squared (m^2^). Blood pressure was measured three times using a validated semi-automated device (The Microlife WatchBP home) with appropriate cuffs in a sitting position after at least 5 min rest. The mean of the last two blood pressure measurements was used in the analyses (mmHg). Concentrations of total blood cholesterol, low‐density lipoprotein (LDL) cholesterol and high‐density lipoprotein (HDL) cholesterol were assessed using colorimetric test kits (mmol/L). Fasting plasma glucose concentration (minimum overnight fast of 8 h) was measured using an enzymatic method (hexokinase) in mmol/L. Presence of type 2 diabetes (T2D) was defined using the WHO diagnostic criteria (fasting glucose ≥ 7.0 mmol/L, or current use of medication prescribed to treat T2D or self‐reported T2D). CRP was measured using the high sensitivity immunoturbidimetric assay in mg/L. For baseline characteristics, CRP was categorized according to American Heart Association (AHA) categories: <1 mg/L = low, 1–3 mg/L = borderline, >3 mg/L = elevated. All biochemical analyses were performed in Berlin with an ABX Pentra 400 chemistry analyser (ABX Pentra; Horiba ABX, Germany).

### DNAm processing, profiling and quality control

Assessment of the epigenetic profiles, its processing, and quality control within the RODAM study were described previously^15^. In brief, bisulfite conversion of DNA was conducted with the Zymo EZ DNA MethylationTM kit. The converted DNA was amplified and hybridized on the Infinium® HumanMethylation450 BeadChip, which quantifies DNAm levels of approximately 485,000 CpG sites. Quality control was performed using the *MethylAid* package in R (*version 1.4.0.)*. Functional normalization (which uses the internal control probes present on the array to infer between-array technical variation) was applied using *minfi* package (*version 3.1.0*)^53,54^. Probes annotated to the X, and Y chromosomes, known to involve cross hybridization or to involve a (common) SNP were removed from the dataset, resulting in a total set of 429,459 CpG sites. Blood cell mixture estimation was based on the method described by Houseman et al. Bioconductor *sva* package was used to construct surrogate variables for removal of unwanted variation^55^.

### Statistical analysis

Statistical analysis was carried out using R and Bioconductor packages. Summary statistics were presented as proportions for categorical variables and as means (with standard deviations) or as medians (with interquartile ranges). Linear regression analyses were performed to determine associations between DNAm and CRP (continuous variable) using the *minfi* package (*version 1.34.0*, DNAm are the dependent variable). Age, sex, alcohol consumption, smoking, estimated cell types and technical effects (hybridization batch and array position) and surrogate variables were included as covariates in all models. We also included BMI and T2D as covariates in the base models to account for previous RODAM reports, which showed an enrichment for obesity and T2D^15,16,56^. Inflation model fitting was evaluated using a QQ-plot (Supplementary Fig. 8). False discovery rate (FDR) was used to correct for multiple testing. A 5% FDR was considered statistically significant. For all DMP analyses, M values were calculated as the log2 ratio of the intensities of methylated CpG site versus unmethylated CpG site. Significant differences were determined based on M values, while beta values were used for visualization^57^. To detect DMRs, we fitted models similar to DMP analyses using *bumphunter* with a cut-off of 0.0066 (which limits the analysis to 100 candidate regions and 0.66% difference in delta-beta values between candidate probes) and 1000 permutations^54^. We considered DMRs with ≥three adjacent probes and 5% FDR as statistically significant.

### Post-hoc analyses

We performed multiple post-hoc analyses to ascertain validity of our findings. First, we performed an in-silico replication. To achieve this, we referred to the previous EWAS meta-analysis on CRP and performed an independent statistical analysis on these previously reported DMPs, employing linear regression models like our main DMP analyses^7^. We assumed statistical significance at a nominal *p*-value of 0.05 (two-tailed).

Second, we carried out sensitivity analyses on obesity and T2D. Since DNAm profiling in the primary RODAM study was based on obese or T2D case-control status, we performed a sensitivity analysis to determine the influence of BMI and T2D in the current analysis^15,16^. To achieve this, we repeated the statistical procedure applied in the main analysis but excluded BMI and T2D as covariates in the linear regression models. In turn, we assessed whether delta-beta values and *p*-values ascribed to statistically significant DMPs in main analysis were strongly correlated with those (delta-beta values and *p*-values) in sensitivity analyses (linear models excluding BMI and T2D as covariates). A Pearson’s correlation coefficient (r) >0.80 was considered strong correlation. Additionally, we looked to previous EWAS on obesity and T2D in the same study population and ascertained whether our statistically significant DMPs were already reported in those two previous studies^15,16^.

Third, we performed sensitivity analyses on location of residence. Considering that Ghanaians sampled in our study were resident on two different continents (in Ghana and in Europe). The environment in Europe differs to that in Ghana (i.e., pollution, microbes, diet etc.), which might distinctively affect inflammatory patterns in Ghanaian’s resident in Ghana as compared to Ghanaians resident in Europe. We therefore assessed the influence of location of residence on our findings. We first applied similar statistical procedures from the main analysis to Ghanaians resident in Ghana and Ghanaians resident in Europe separately. We then compared the effect sizes (delta-beta values) between the two groups for the DMPs that were statistically significant in the main analysis. Next, we repeated the statistical procedure applied in the main analysis and included location of residence as a covariate in the linear regression models. In turn, we assessed whether delta-beta values and *p*-values for the statistically significant DMPs in the main analysis were strongly correlated with those (delta-beta values and *p*-values) from sensitivity analyses (linear models including location of residence as a covariate). A Pearson’s correlation coefficient (r) >0.80 was considered strong correlation.

Fourth, we examined DNAm differences at CRP levels ≤10 mg/L. With regards to chronic inflammation, CRP concentrations ≤10 mg/L are usually seen in healthy adults without chronic diseases, while those >10 mg/L are mostly seen in those with chronic diseases^42,43^. Since we had included CRP concentrations up to 40 mg/L in our main analysis, we also wanted to ascertain whether DNAm variations were also detectable at CRP levels ≤10 mg/L in our study population. We therefore performed a subset analysis on participants with CRP concentrations ≤10 mg/L employing similar statistical procedures as in the main analysis.

Fifth, we investigated the link between DNAm variations in CRP and estimated CVD risk. To achieve this, we selected statistically significant DMPs from the main analysis and investigated their associations with 10-year predicted CVD risk using linear regression models. Ten-year CVD risk was estimated using the American College of Cardiology/American Heart Association atherosclerotic cardiovascular disease (ACC/AHA ASCVD) risk score as previously applied in RODAM study^58^. The risk score is used among persons aged 40–79 years, without prior history of CVD, using an algorithm that combines age, sex, use of antihypertensive medication, systolic blood pressure, presence of T2D, total cholesterol, HDL cholesterol and smoking status^59^. A score of >7.5% is considered to be an elevated risk of developing a CVD in the next 10 years based on the prior work by Goff et al.^59^. We assumed statistical significance at 5% FDR. Variables that were already part of the CVD risk score were not adjusted for in our linear regression models.

Sixth, we ascertained biological relevance of findings with respect to gene expression, and links to inflammation and CVD in public databases. We first evaluated the link between statistically significant DMPs and gene expression in the IMETHYL database^50^. IMETHYL provides whole-DNA, whole-genome and whole-transcriptome data for normal CD4+ T-lymphocytes, monocytes and neutrophils collected from ~100 healthy subjects. Next, we assessed whether genes annotated to statistically significant DMPs were enriched to discrete pathways in the KEGG and GO databases using *MissMethyl* package at 5% FDR. Finally, we searched in GWAS catalog, GeneCards and EWAS catalog to determine whether genes annotated to statistically significant DMPs were linked to inflammation or CVD traits.

### Reporting summary

Further information on research design is available in the Nature Research Reporting Summary linked to this article.

## Supplementary information

Supplementary Information

Reporting Summary

## Data Availability

Individual participant data from the RODAM study used in the current analyses were deposited to the European Genome-Phenome Archive (https://ega-archive.org/) in a deidentified or anonymised format. Accession number is EGAS00001005162. Data will be shared with researchers submitting a research proposal and requesting access to data. Data will be made available for analyses as approved by the data access committee.
