# Supplementary material for: Genome-wide DNA methylation analysis on C-reactive protein among Ghanaians suggests molecular links to the emerging risk of cardiovascular diseases
Source: NPJ Genom Med. 2021 Jun 11;6:46. doi: 10.1038/s41525-021-00213-9 (PMC8196035; doi:10.1038/s41525-021-00213-9)
Supplement: Supplementary file 1 — Supplementary Information [file 41525_2021_213_MOESM1_ESM.pdf]

Supplementary data contents

Supplementary Figure 1: Differential DNA methylation in cg02338947 with respect to CRP levels...... 1

Supplementary Table 1: Candidate list of CpGs identified in previous EWAS meta-analysis of CRP.......3

Supplementary Table 2: Candidate probes associated with CRP (mg/L) that successfully replicated in the RODAM (Ghanaians) 8

Supplementary Figure 2: Sensitivity analyses on Body Mass Index (BMI) and Type 2 Diabetes (T2D) ….10

Supplementary Table 3: Differences in effect sizes of DNA methylation variations with CRP between Ghanaians resident in Europe vs Ghanaians resident in Ghana........12

Supplementary Figure 3: Sensitivity analyses on location of residence (Ghanaians resident in Ghana vs Ghanaians resident in Europe. 14

Supplementary Figure 4: Manhattan plot of DMPs associated with CRP ≤ 10 mg/L among Ghanaians….18

Supplementary Figure 5: Inflation modelling with QQ plots for CRP levels ≤ 10 mg/L 19

Supplementary Table 4: Differentially methylated position (DMP) associated with CRP at levels ≤ 10 mg/L 20

Supplementary Table 5: Pathway analysis of genome wide significant DMPs in the GO (Gene Ontology) database. 25

Supplementary Table 6: Pathway analysis of genome wide significant DMPs in the KEGG (Kyoto Encyclopedia of Genes and Genomes) database. 27

Supplementary Table 7: Gene function and associated phenotypes in EWAS catalog........28

Supplementary Table 8: Gene function and associated phenotypes in GWAS catalog and GeneHancer database........33

Supplementary Figure 6: Flow chart of participation 38

Supplementary Figure 7: Distribution of CRP in the final sample........40

Supplementary Figure 8: Inflation modelling with QQ plots in the main analysis 42

Supplementary Figure 1. Differential DNA methylation in cg02338947 (3'UTR of FAM124B gene) with respect to CRP levels.

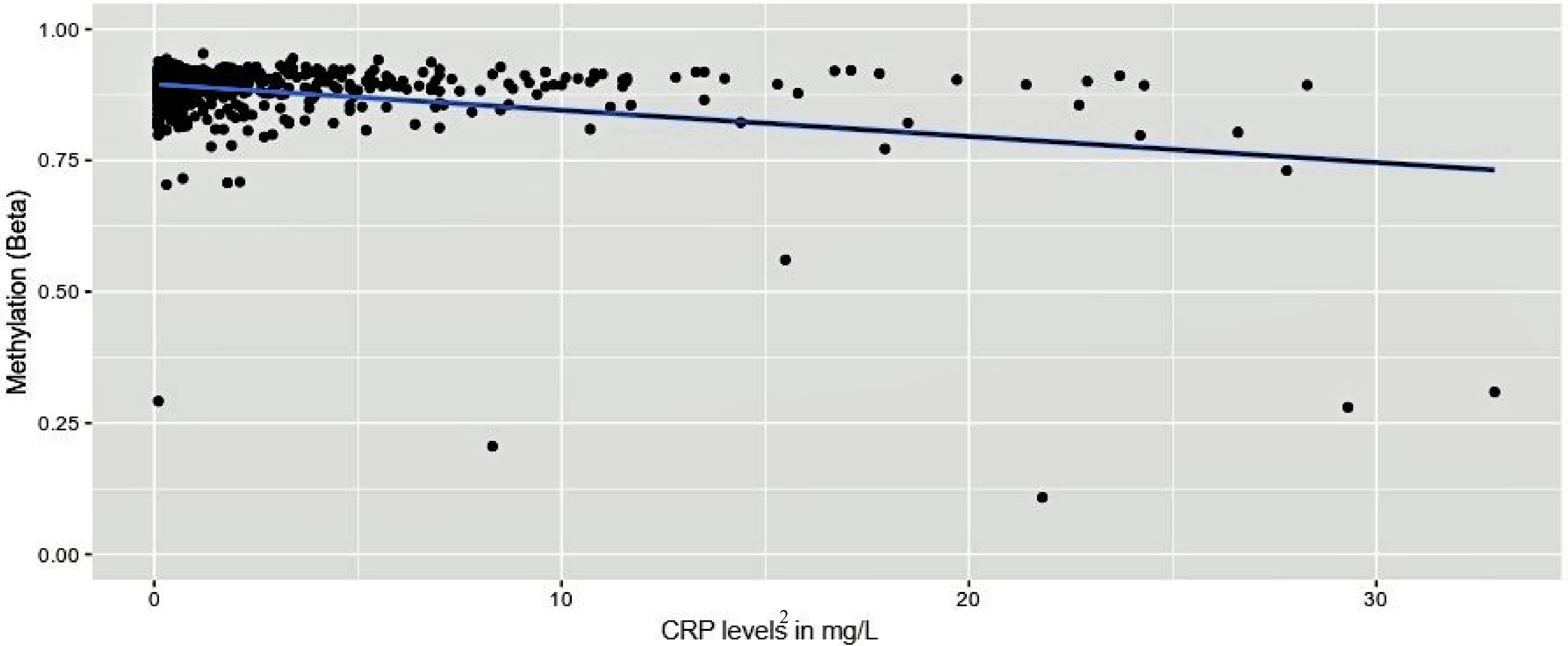
1.00
0.75
0.50
0.25
0.00
Methylation (Beta)
0
10
20
30
CRP levels in mg/L

Supplementary Table 1.Candidate list of CpGs identified in previous EWAS meta-analysis of CRP

Ligthart S, Marzi C, Aslibekyan S, Mendelson MM, Conneely KN, Tanaka T, Colicino E, Waite LL, Joehanes R, Guan W, Brody JA. DNA methylation signatures of chronic low-grade inflammation are associated with complex diseases. Genome biology. 2016 Dec;17(1):1-5.

EWAS meta-analysis of CRP (mg/L) in Europeans only

MarkerName	Chr	Position	Gene	Effect	P value
cg10636246	1	159046973	*AIM2*	-0.0069	2.53E-27
cg17501210	6	166970252	*RPS6KA2*	-0.0065	2.06E-26
cg02650017	17	47301614	*PHOSPHO1*	-0.0021	4.87E-25
cg12992827	3	101901234		-0.0057	9.73E-22
cg16936953	17	57915665	*TMEM49*	-0.0077	3.74E-21
cg19821297	19	12890029	*HOOK2*	-0.0051	5.19E-21
cg07573872	19	1126342	*SBNO2*	-0.0052	1.24E-20
cg26470501	19	45252955	*BCL3*	-0.0045	2.85E-20
cg12054453	17	57915717	*TMEM49*	-0.0082	6.96E-20
cg18608055	19	1130866	*SBNO2*	-0.0043	1.94E-19
cg06192883	15	52554171	*MYO5C*	0.0045	2.29E-19
cg18181703	17	76354621	*SOCS3*	-0.0053	2.13E-18
cg18942579	17	57915773	*TMEM49*	-0.0056	4.77E-16
cg14476101	1	120255992	*PHGDH*	-0.0068	5.29E-16
cg22304262	19	47287778	*SLC1A5*	-0.0045	6.55E-16
cg02711608	19	47287964	*SLC1A5*	-0.0034	8.42E-16
cg02716826	9	33447032	*SUGT1P1*	-0.0037	1.32E-15
cg19769147	14	105860954	*PACS2*	0.0029	1.51E-15
cg09018739	16	57180107	*CPNE2*	0.0035	1.64E-15
cg20995564	2	145172035	*ZEB2*	-0.0051	2.04E-15
cg02734358	4	90227074	*GPRIN3*	-0.0048	3.09E-15
cg23842572	17	17030253	*MPRIP*	0.0032	3.91E-15
cg07094298	4	2748026	*TNIP2*	-0.0056	4.76E-15
cg13165240	17	3715743	*C17orf85*	0.0037	1.58E-14
cg01059398	3	172235808	*TNFSF10*	-0.0042	4.51E-14
cg25217710	1	156609523		0.0026	5.11E-14
cg00490406	1	159046773	*AIM2*	-0.0058	9.35E-14
cg01101459	1	234871477		0.0035	1.15E-13
cg06690548	4	139162808	*SLC7A11*	-0.0048	1.21E-13
cg26227957	1	19547285	*KIAA0090*	0.0033	2.38E-13
cg25132241	14	92396859	*FBLN5*	0.0027	2.86E-13
cg05279866	8	37378355		0.0030	3.28E-13
cg02003183	14	103415882	*CDC42BPB*	0.0047	3.59E-13
cg26804423	7	8201134	*ICA1*	0.0027	3.87E-13
cg22959742	10	13913931	*FRMD4A*	0.0042	4.12E-13
cg11024682	17	17730094	*SREBF1*	0.0030	5.04E-13
cg15357118	2	128927972	*UGGT1*	0.0035	5.77E-13
cg18125510	14	100841768	*WARS*	-0.0033	6.42E-13
cg13585930	10	72027357	*NPFFR1*	-0.0037	1.42E-12
cg00309945	1	22148926	*LDLRAD2*	0.0035	1.47E-12
cg09481056	16	2339260	*ABCA3*	0.0028	1.57E-12
cg12555233	15	91455366	*MAN2A2*	0.0023	2.15E-12
cg03957124	6	37016869		-0.0030	3.13E-12
cg10919522	14	74227441	*C14orf43*	-0.0035	3.28E-12
cg05248321	14	20898128	*KLHL33*	0.0034	3.36E-12
cg15114651	19	47289410	*SLC1A5*	-0.0025	4.55E-12
cg14849578	12	125282480	*SCARB1*	0.0034	4.90E-12
cg12053291	12	125282342	*SCARB1*	0.0029	5.99E-12
cg19588519	10	125817817		0.0029	6.20E-12
cg15901722	5	175974973	*PCDH24*	-0.0032	6.22E-12
cg02481950	16	21665002	*METTL9*	0.0022	7.84E-12

cg04987734	14	103415873	*CDC42BPB*	0.0041	8.40E-12
cg13300580	1	27440539	*SLC9A1*	0.0023	8.52E-12
cg07078732	2	30505165		0.0023	1.22E-11
cg15551881	9	123688715	*TRAF1*	0.0039	4.62E-11
cg27023597	17	57918262	*MIR21*	-0.0050	5.02E-11
cg05575921	5	373378	*AHRR*	-0.0059	5.44E-11
cg27469606	19	1154485	*SBNO2*	-0.0020	5.62E-11
cg17178175	2	178109973	*NFE2L2*	-0.0037	7.46E-11
cg08289839	13	111318640	*CARS2*	0.0027	8.76E-11
cg05399785	1	3564031	*WDR8*	0.0029	9.31E-11
cg11832534	1	3563998	*WDR8*	0.0024	9.97E-11
cg12728588	1	36025489	*NCDN*	0.0019	1.15E-10
cg10241823	17	841205	*NXN*	0.0035	1.29E-10
cg20671910	1	151262619	*ZNF687*	0.0025	2.30E-10
cg01243823	16	50732212	*NOD2*	-0.0038	2.31E-10
cg18860310	4	87752504	*SLC10A6*	-0.0031	2.96E-10
cg11183227	15	91455407	*MAN2A2*	0.0030	3.02E-10
cg23759710	2	42990957	*OXER1*	0.0019	3.50E-10
cg01409343	17	57915740	*TMEM49*	-0.0037	3.56E-10
cg20697417	1	41786797		0.0028	3.99E-10
cg02976843	17	2843257	*RAP1GAP2*	0.0029	4.34E-10
cg21429551	7	30635762	*GARS*	-0.0069	4.42E-10
cg03725309	1	109757585	*SARS*	-0.0022	4.54E-10
cg18217136	20	36157651	*BLCAP*	0.0020	5.26E-10
cg07626482	19	47289503	*SLC1A5*	-0.0027	5.63E-10
cg26955383	10	105218660	*CALHM1*	0.0029	5.70E-10
cg14237301	16	28506477	*APOB48R*	0.0041	5.77E-10
cg04255937	12	122269675	*SETD1B*	0.0026	6.21E-10
cg13033858	12	109248326	*SSH1*	-0.0036	6.58E-10
cg23886355	13	113662042	*MCF2L*	0.0026	6.80E-10
cg07037944	15	64290807	*DAPK2*	-0.0024	7.99E-10
cg01418351	4	1294432	*MAEA*	0.0022	8.51E-10
cg23761815	10	73083123	*SLC29A3*	0.0022	8.86E-10
cg25653947	8	144443217		0.0020	9.24E-10
cg08548559	22	31686097	*PIK3IP1*	-0.0038	9.94E-10
cg26610247	8	142297175		0.0029	1.07E-09
cg11376147	11	57261198	*SLC43A1*	-0.0016	1.08E-09
cg08255481	16	88103035	*BANP*	0.0025	1.12E-09
cg16246545	1	120255941	*PHGDH*	-0.0042	1.23E-09
cg27050612	17	46133198	*NFE2L1*	-0.0019	1.30E-09
cg18513344	3	195531298	*MUC4*	-0.0020	1.39E-09
cg07021906	16	87866833	*SLC7A5*	0.0032	1.45E-09
cg05400196	6	42272048	*TRERF1*	0.0021	1.63E-09
cg16536330	8	29957344	*LEPROTL1*	-0.0014	1.69E-09
cg15721584	3	181326755	*SOX2OT*	0.0055	1.71E-09
cg03068497	7	30635838	*GARS*	-0.0064	1.76E-09
cg06126421	6	30720080		-0.0052	1.80E-09
cg09610644	3	197249274	*BDH1*	0.0032	1.89E-09
cg00851028	1	234905772		0.0023	1.95E-09
cg24174557	17	57903544	*TMEM49*	-0.0038	1.97E-09
cg13059136	11	2986541	*SNORA54*	0.0032	2.16E-09
cg14753356	6	30720108		-0.0032	2.20E-09
cg05316065	8	130799007	*GSDMC*	-0.0027	2.26E-09
cg27516100	6	30624520	*DHX16*	0.0020	2.38E-09
cg24925163	2	128458248	*SFT2D3*	0.0022	2.40E-09
cg02464912	14	64319543	*SYNE2*	-0.0009	2.42E-09
cg04523589	3	48265146	*CAMP*	0.0022	2.49E-09
cg22908922	14	105855008	*PACS2*	0.0023	2.64E-09
cg24707889	21	46341304	*ITGB2*	0.0032	2.71E-09

cg27307975	15	58797839	*LIPC*	-0.0019	2.75E-09
cg01445100	16	88103339	*BANP*	0.0020	2.81E-09
cg16305292	8	142219965		0.0024	2.82E-09
cg05232694	20	48809539		-0.0053	3.02E-09
cg06484123	19	53107200		0.0054	3.49E-09
cg01127300	22	38614796		-0.0044	3.90E-09
cg01538969	6	30624636	*DHX16*	0.0027	4.09E-09
cg11136886	1	33609509		0.0022	4.16E-09
cg03963853	16	4732369	*MGRN1*	0.0023	4.38E-09
cg08539067	3	49395985	*GPX1*	-0.0015	4.58E-09
cg17980786	3	32933637	*TRIM71*	0.0026	4.58E-09
cg24259291	20	47874072	*ZNFX1*	0.0020	4.66E-09
cg26599989	11	1297087	*TOLLIP*	0.0025	4.95E-09
cg11132913	1	59285109		0.0026	5.21E-09
cg25325512	6	37142220	*PIM1*	-0.0031	5.31E-09
cg00812761	4	53799391	*SCFD2*	0.0025	5.60E-09
cg26680989	16	85560739		0.0024	5.62E-09
cg21766592	19	47288066	*SLC1A5*	-0.0019	5.64E-09
cg27637521	17	76355202	*SOCS3*	-0.0016	5.69E-09
cg27115863	22	37921640		-0.0030	5.77E-09
cg26846781	17	61620942	*KCNH6*	0.0018	5.99E-09
cg04389058	3	57041402	*ARHGEF3*	-0.0030	6.77E-09
cg03290827	17	75180760	*SEC14L1*	0.0029	6.93E-09
cg22448090	1	200978598	*KIF21B*	0.0019	7.06E-09
cg12298872	1	228352449	*C1orf69*	0.0026	7.84E-09
cg11064521	15	69606134	*PAQR5*	0.0018	7.90E-09
cg05168229	13	45390049		-0.0022	8.06E-09
cg00159243	12	109023799	*SELPLG*	-0.0026	8.22E-09
cg22103219	7	101934892	*SH2B2*	-0.0032	8.48E-09
cg15310871	8	20077936	*ATP6V1B2*	0.0022	8.63E-09
cg07248377	16	4732406	*MGRN1*	0.0032	9.67E-09
cg02909097	17	2843206	*RAP1GAP2*	0.0025	9.75E-09
cg00587301	6	156717406		0.0019	1.00E-08
cg18120259	6	43894639	*LOC100132354*	-0.0027	1.00E-08
cg08423142	15	59588622	*MYO1E*	-0.0016	1.03E-08
cg25754958	1	202130692	*PTPN7*	-0.0032	1.03E-08
cg01588592	1	157069325	*ETV3L*	0.0023	1.10E-08
cg02448796	1	6101339	*KCNAB2*	0.0031	1.39E-08
cg09876440	3	10325981	*GHRLOS*	-0.0045	1.42E-08
cg17572056	3	195947062	*OSTalpha*	0.0024	1.66E-08
cg13781414	9	138951648	*NACC2*	-0.0021	1.67E-08
cg15020801	17	46022809	*PNPO*	0.0024	1.67E-08
cg10508317	17	76355146	*SOCS3*	-0.0020	1.69E-08
cg08594651	11	47415397		-0.0022	1.77E-08
cg16755922	17	80536214	*FOXK2*	0.0035	1.79E-08
cg16097041	1	154965544	*FLAD1*	0.0023	1.82E-08
cg03128029	2	203143288	*NOP58*	-0.0027	1.90E-08
cg04465154	8	9045558		-0.0031	1.94E-08
cg06164260	3	187454439	*BCL6*	-0.0019	2.05E-08
cg09007354	1	54100163	*GLIS1*	0.0023	2.06E-08
cg18330338	5	9047211	*SEMA5A*	0.0027	2.12E-08
cg02050917	1	2173571	*SKI*	0.0024	2.15E-08
cg17237086	22	40814966	*MKL1*	0.0021	2.21E-08
cg01332882	20	47874155	*ZNFX1*	0.0032	2.26E-08
cg05131266	14	91591888	*C14orf159*	0.0028	2.52E-08
cg10409253	7	5692709	*RNF216*	0.0023	2.52E-08
cg04583842	16	88103117	*BANP*	0.0035	2.55E-08
cg04202511	16	68117991	*NFATC3*	0.0025	2.56E-08
cg26250129	17	79239903	*SLC38A10*	0.0021	2.63E-08

cg06017212	17	1478463	*SLC43A2*	0.0019	2.73E-08
cg18062721	3	11643427	*VGLL4*	0.0041	2.73E-08
cg20451986	11	133928302		0.0033	2.91E-08
cg21990700	12	7260776	*LOC283314*	-0.0028	2.94E-08
cg10589813	20	48809978		-0.0026	3.14E-08
cg12315466	2	233923814	*INPP5D*	0.0019	3.19E-08
cg22749855	17	76353952	*SOCS3*	-0.0024	3.22E-08
cg24296397	3	49692537	*BSN*	0.0024	3.29E-08
cg03699074	16	88849875	*FAM38A*	-0.0024	3.71E-08
cg25368647	5	176736591	*MXD3*	-0.0033	3.89E-08
cg02341197	21	34185927	*C21orf62*	0.0030	3.92E-08
cg01833890	12	2564063	*CACNA1C*	0.0017	4.03E-08
cg16739178	16	85470674		0.0024	4.03E-08
cg24002003	15	101668143		-0.0024	4.04E-08
cg15342087	6	30720209		-0.0019	4.10E-08
cg12269535	6	43142014	*SRF*	-0.0028	4.39E-08
cg23172671	1	203482523		0.0035	4.41E-08
cg03963219	2	84709147		0.0018	4.70E-08
cg08507178	22	50357265	*PIM3*	-0.0019	5.09E-08
cg25392060	8	142297121		0.0025	5.60E-08
cg04321224	21	46341380	*ITGB2*	0.0026	5.62E-08
cg23866916	19	1155738	*SBNO2*	-0.0018	5.63E-08
cg16395997	1	3562798	*WDR8*	0.0028	5.76E-08
cg27184903	15	29285727	*APBA2*	0.0024	5.84E-08
cg20510033	1	8960134		-0.0026	6.05E-08
cg22688566	17	27459835	*MYO18A*	0.0025	6.06E-08
cg09940677	14	103415458	*CDC42BPB*	0.0019	6.45E-08
cg19445588	12	116904641		0.0023	6.54E-08
cg05673882	5	74862702	*POLK*	-0.0038	6.89E-08
cg18663307	21	46341389	*ITGB2*	0.0029	6.98E-08
cg15553397	1	76695186	*ST6GALNAC3*	0.0021	7.14E-08
cg12535090	11	20071677	*NAV2*	0.0021	7.25E-08
cg05897122	4	53112707		-0.0035	7.46E-08
cg26547058	8	142243143		0.0022	7.46E-08
cg02203067	16	87866800	*SLC7A5*	0.0024	7.62E-08
cg09421562	17	56357994	*MPO*	-0.0023	7.64E-08
cg01242348	14	23586886	*CEBPE*	0.0015	8.33E-08
cg17907003	1	117533414		-0.0027	8.40E-08
cg08362785	22	40814878	*MKL1*	0.0019	8.47E-08
cg24048338	8	43046482	*HGSNAT*	-0.0023	8.98E-08
cg09182678	22	50328711		-0.0016	9.02E-08
cg17260706	11	118782879	*BCL9L*	-0.0020	9.63E-08
cg12955084	19	41886265	*TMEM91*	0.0024	9.76E-08
cg00227093	5	149979838	*SYNPO*	-0.0026	9.80E-08
cg23740758	6	11324433	*NEDD9*	0.0022	9.99E-08
cg18772573	17	71257980	*CPSF4L*	0.0022	1.01E-07
cg26663590	16	28959310		0.0030	1.04E-07
cg18476766	2	240529742		0.0032	1.06E-07
cg02059519	9	137250935	*RXRA*	0.0020	1.10E-07

EWAS meta-analysis of CRP in Europeans and African Americans combined (duplicates from Europeans only analysis were removed)

MarkerName	Chr	Position	Gene	Effect	P.value
cg00711496	19	50191497	*C19orf76*	-0.0042	9.95E-09
cg01015663	1	23729692	*TCEA3*	-0.0029	1.59E-08
cg01243312	2	136422981	*MIR128-1*	0.0039	5.89E-10
cg01671681	3	155421735	*PLCH1*	-0.003	1.01E-07
cg01799015	19	707791	*PALM*	-0.0025	4.39E-08
cg02068690	2	25600451	*DTNB*	0.0019	6.96E-08

cg02212836	6	6589075	*LY86*	0.002	7.15E-08
cg02538248	16	75045643	*ZNRF1*	-0.0036	5.09E-08
cg02787737	11	133928346	*NA*	0.0031	1.08E-07
cg03055671	3	172231528	*TNFSF10*	-0.0042	8.36E-08
cg03497652	16	4751569	*ANKS3*	0.0033	7.10E-08
cg03650189	19	10405083	*ICAM5*	0.0049	4.84E-08
cg03940776	6	158490013	*SYNJ2*	-0.0018	7.98E-08
cg03998636	13	111210121	*RAB20*	-0.0023	2.68E-08
cg04508739	14	57263369	*NA*	0.0028	7.81E-08
cg06092244	2	48796151	*STON1-GTF2A1L*	-0.0025	1.46E-08
cg06291107	20	36157675	*BLCAP*	0.0019	6.59E-08
cg06647068	12	104853274	*CHST11*	-0.0033	1.90E-08
cg06946797	16	11422409	*NA*	-0.003	4.07E-08
cg07069636	16	30671749	*NA*	-0.0022	9.03E-08
cg07793033	16	85256423	*NA*	0.0028	2.82E-08
cg07960624	8	119208486	*SAMD12*	-0.004	4.63E-08
cg08352115	17	66356057	*ARSG*	0.0026	3.65E-09
cg08645860	16	85969301	*NA*	0.0028	6.02E-09
cg09476997	16	2087932	*SLC9A3R2*	0.0022	9.17E-08
cg10345404	8	27187123	*PTK2B*	0.0016	3.09E-08
cg10472711	7	797592	*HEATR2*	-0.0029	2.57E-08
cg10922280	16	68034227	*DPEP2*	0.0022	1.44E-09
cg11032810	4	141057042	*MAML3*	-0.0017	1.10E-07
cg11849692	10	103875969	*LDB1*	0.0019	2.60E-08
cg12458003	1	204960826	*NFASC*	0.0023	8.07E-08
cg12620005	1	51779655	*TTC39A*	-0.0016	1.04E-07
cg12644285	15	93570953	*CHD2*	-0.003	5.06E-08
cg13274938	17	38493822	*RARA*	0.002	6.75E-08
cg13328614	X	48775123	*PIM2*	-0.0038	1.30E-08
cg15011409	19	10405226	*ICAM5*	0.0049	5.36E-08
cg15809077	2	112462079	*NA*	-0.0012	4.41E-08
cg16292768	8	27467783	*CLU*	-0.0028	1.61E-08
cg16734549	17	43488551	*ARHGAP27*	0.0021	8.38E-08
cg17515347	1	159047163	*AIM2*	-0.0054	1.28E-08
cg18995788	8	62673444	*NA*	-0.002	5.06E-08
cg19137806	10	134362170	*INPP5A*	0.0027	4.08E-08
cg19695507	10	13526193	*BEND7*	0.0029	8.36E-08
cg20045320	11	319555	*NA*	-0.005	1.19E-08
cg21106695	14	97370064	*NA*	0.003	7.92E-08
cg21190595	11	3071167	*CARS*	-0.0021	5.72E-08
cg22069247	2	232393256	*NMUR1*	0.0025	1.15E-07
cg22488164	12	14716910	*PLBD1*	0.0025	7.35E-08
cg22690339	6	38249061	*BTBD9*	0.0038	8.49E-09
cg23570810	11	315102	*IFITM1*	-0.004	6.25E-08
cg23801885	6	34458902	*PACSIN1*	0.0019	7.64E-08
cg23966214	17	48203188	*SAMD14*	-0.0018	1.57E-08
cg24405567	15	70787565	*NA*	-0.0031	3.79E-08
cg24678869	1	153919638	*DENND4B*	0.0017	9.05E-08
cg24859433	6	30720203	*NA*	-0.0019	8.20E-08
cg25114611	6	35696870	*FKBP5*	-0.0021	7.81E-08
cg25130381	1	27440721	*SLC9A1*	0.002	3.14E-08
cg25580581	10	3853668	*NA*	-0.0031	2.37E-08
cg25739715	22	30663881	*OSM*	-0.0012	1.84E-08
cg25921813	3	194797138	*C3orf21*	0.0019	6.62E-08
cg26581729	9	139939792	*NPDC1*	-0.0031	5.82E-09
cg27269962	7	127540997	*SND1*	0.0019	9.44E-08

Supplementary Table 2. Candidate probes associated with CRP (mg/L) that successfully replicated in the RODAM study (Ghanaians)

replication statistical analyses was at nominal $p<0.05$

				RODAM study		Previous Meta-analysis	
MarkerName	Chr	Position	Nearest Gene	Delta-beta value	P-value	Delta-beta value	P value
cg18608055	chr19	1130866	*SBNO2*	-0.0017	2.05E-06	-0.0043	1.94E-19
cg19821297	chr19	12890029	*HOOK2*	-0.0019	2.68E-05	-0.0051	5.19E-21
cg00490406	chr1	159046773	*AIM2*	-0.0028	6.33E-05	-0.0058	9.35E-14
cg10636246	chr1	159046973	*AIM2*	-0.0019	7.19E-05	-0.0069	2.53E-27
cg17515347	chr1	159047163	*AIM2*	-0.0031	9.01E-05	-0.0054	1.28E-08
cg18860310	chr4	87752504	*SLC10A6*	-0.0014	9.29E-05	-0.0031	2.96E-10
cg13585930	chr10	72027357	*NPFFR1*	-0.0017	1.20E-04	-0.0037	1.42E-12
cg05316065	chr8	130799007	*GSDMC*	-0.0015	1.88E-04	-0.0027	2.26E-09
cg09421562	chr17	56357994	*MPO*	-0.0014	1.94E-04	-0.0023	7.64E-08
cg00227093	chr5	149979838	*SYNPO*	-0.0017	2.96E-04	-0.0026	9.80E-08
cg24002003	chr15	101668143	*Intergenic*	-0.0011	3.48E-04	-0.0024	4.04E-08
cg20995564	chr2	145172035	*ZEB2*	-0.0022	4.14E-04	-0.0051	2.04E-15
cg13781414	chr9	138951648	*NACC2*	-0.0012	5.36E-04	-0.0021	1.67E-08
cg26581729	chr9	139939792	*NPDC1*	-0.0014	5.45E-04	-0.0031	5.82E-09
cg02003183	chr14	103415882	*CDC42BPB*	0.0017	5.89E-04	0.0047	3.59E-13
cg07573872	chr19	1126342	*SBNO2*	-0.0019	7.97E-04	-0.0052	1.24E-20
cg02650017	chr17	47301614	*PHOSPHO1*	-0.0005	1.00E-03	-0.0021	4.87E-25
cg22749855	chr17	76353952	*SOCS3*	-0.0014	1.12E-03	-0.0024	3.22E-08
cg06092244	chr2	48796151	*STON1*	-0.0012	2.38E-03	-0.0025	1.46E-08
cg03998636	chr13	111210121	*RAB20*	-0.0010	2.66E-03	-0.0023	2.68E-08
cg15809077	chr2	112462079	*Intergenic*	-0.0007	2.68E-03	-0.0012	4.41E-08
cg18181703	chr17	76354621	*SOCS3*	-0.0014	2.70E-03	-0.0053	2.13E-18
cg03725309	chr1	109757585	*SARS*	-0.0009	2.76E-03	-0.0022	4.54E-10
cg02734358	chr4	90227074	*GPRIN3*	-0.0014	2.89E-03	-0.0048	3.09E-15
cg12269535	chr6	43142014	*SRF*	-0.0013	3.42E-03	-0.0028	4.39E-08
cg15901722	chr5	175974973	*PCDH24*	-0.0011	3.44E-03	-0.0032	6.22E-12
cg11024682	chr17	17730094	*SREBF1*	0.0010	3.58E-03	0.003	5.04E-13
cg12992827	chr3	101901234	*Intergenic*	-0.0012	4.34E-03	-0.0057	9.73E-22
cg03650189	chr19	10405083	*ICAM5*	0.0017	4.42E-03	0.0049	4.84E-08
cg06647068	chr12	104853274	*CHST11*	-0.0015	4.59E-03	-0.0033	1.90E-08
cg16395997	chr1	3562798	*WDR8*	0.0013	4.67E-03	0.0028	5.76E-08
cg08423142	chr15	59588622	*MYO1E*	-0.0007	6.27E-03	-0.0016	1.03E-08
cg19695507	chr10	13526193	*BEND7*	0.0008	6.49E-03	0.0029	8.36E-08
cg26470501	chr19	45252955	*BCL3*	-0.0009	6.58E-03	-0.0045	2.85E-20
cg04987734	chr14	103415873	*CDC42BPB*	0.0013	7.19E-03	0.0041	8.40E-12
cg04465154	chr8	9045558	*Intergenic*	-0.0015	7.44E-03	-0.0031	1.94E-08
cg19588519	chr10	125817817	*Intergenic*	0.0008	9.05E-03	0.0029	6.20E-12
cg07793033	chr16	85256423	*Intergenic*	0.0010	9.69E-03	0.0028	2.82E-08
cg05248321	chr14	20898128	*KLHL33*	0.0009	1.01E-02	0.0034	3.36E-12
cg13274938	chr17	38493822	*RARA*	0.0009	1.04E-02	0.002	6.75E-08
cg17501210	chr6	166970252	*RPS6KA2*	-0.0019	1.06E-02	-0.0065	2.06E-26
cg12620005	chr1	51779655	*TTC39A*	-0.0008	1.08E-02	-0.0016	1.04E-07
cg12644285	chr15	93570953	*CHD2*	-0.0012	1.11E-02	-0.003	5.06E-08
cg00159243	chr12	109023799	*SELPLG*	-0.0009	1.14E-02	-0.0026	8.22E-09
cg22959742	chr10	13913931	*FRMD4A*	-0.0010	1.20E-02	0.0042	4.12E-13
cg05673882	chr5	74862702	*POLK*	-0.0011	1.28E-02	-0.0038	6.89E-08
cg10919522	chr14	74227441	*C14orf43*	-0.0011	1.30E-02	-0.0035	3.28E-12
cg26955383	chr10	105218660	*CALHM1*	0.0007	1.94E-02	0.0029	5.70E-10
cg15011409	chr19	10405226	*ICAM5*	0.0015	2.10E-02	0.0049	5.36E-08
cg02538248	chr16	75045643	*ZNRF1*	-0.0013	2.29E-02	-0.0036	5.09E-08
cg03940776	chr6	158490013	*SYNJ2*	-0.0005	2.34E-02	-0.0018	7.98E-08
cg27050612	chr17	46133198	*NFE2L1*	-0.0005	2.77E-02	-0.0019	1.30E-09

cg18942579	chr17	57915773	*TMEM49*	-0.0012	2.78E-02	-0.0056	4.77E-16
cg21990700	chr12	7260776	*LOC283314*	-0.0011	2.98E-02	-0.0028	2.94E-08
cg27023597	chr17	57918262	*MIR21*	-0.0014	3.10E-02	-0.005	5.02E-11
cg18062721	chr3	11643427	*VGLL4*	0.0013	3.37E-02	0.0041	2.73E-08
cg04583842	chr16	88103117	*BANP;BANP*	0.0013	3.52E-02	0.0035	2.55E-08
cg15553397	chr1	76695186	*ST6GALNAC3*	0.0006	3.61E-02	0.0021	7.14E-08
cg01799015	chr19	707791	*PALM*	-0.0008	3.61E-02	-0.0025	4.39E-08
cg07069636	chr16	30671749	*Intergenic*	-0.0006	3.80E-02	-0.0022	9.03E-08
cg13033858	chr12	109248326	*SSH1*	-0.0011	4.02E-02	-0.0036	6.58E-10
cg08548559	chr22	31686097	*PIK3IP1*	-0.0009	4.15E-02	-0.0038	9.94E-10
cg25739715	chr22	30663881	*OSM*	-0.0003	4.26E-02	-0.0012	1.84E-08
cg17178175	chr2	178109973	*NFE2L2*	-0.0010	4.36E-02	-0.0037	7.46E-11
cg16292768	chr8	27467783	*CLU*	-0.0008	4.61E-02	-0.0028	1.61E-08
cg06164260	chr3	187454439	*BCL6*	-0.0005	4.69E-02	-0.0019	2.05E-08

Supplementary Figure 2. Sensitivity analyses on Body Mass Index (BMI) and Type 2 Diabetes (T2D)

a) Pearsons correlations between delta-beta values in linear regression models with and without BMI & T2D as covariates

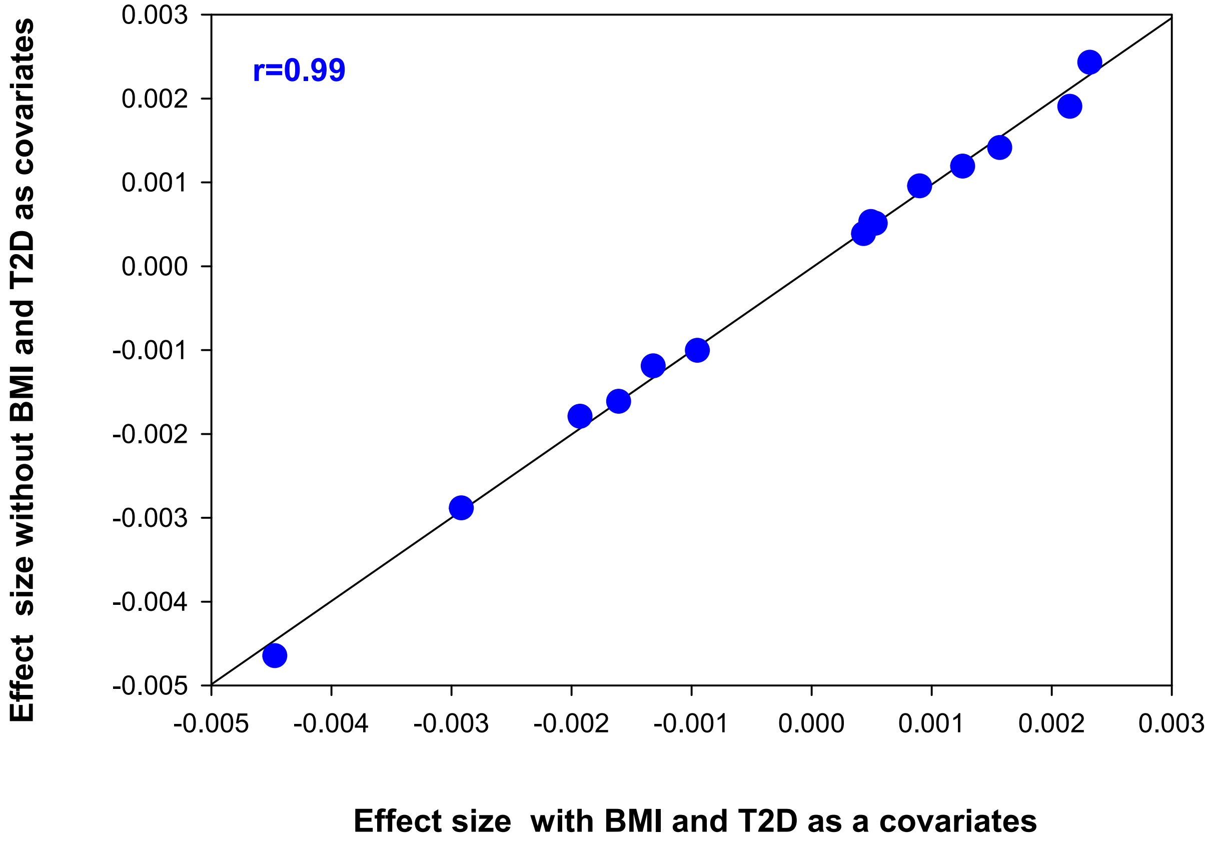

b) Pearsons correlations between P-values in linear regression models with and without BMI & T2D as covariates

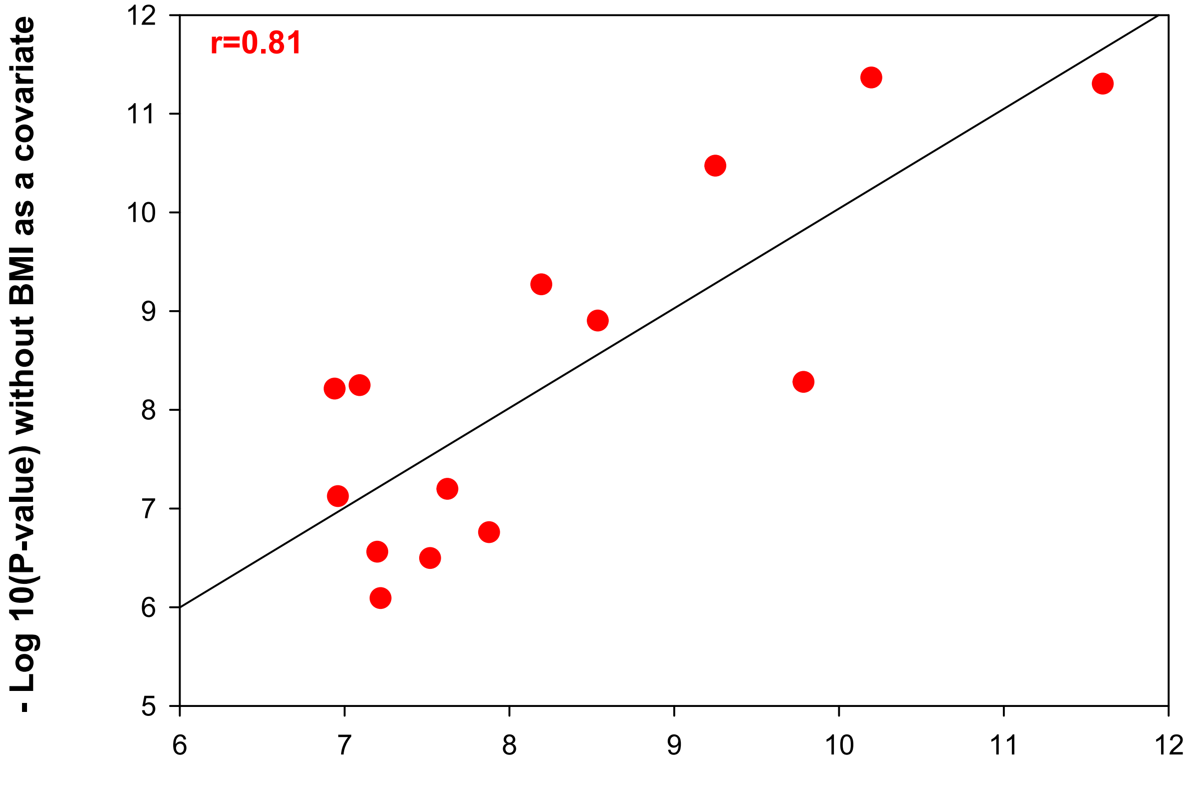

Supplementary Table 3. Differences in effect sizes of DNA methylation variations with CRP between Ghanaians resident in Europe vs Ghanaians resident in Ghana.

				Ghanaians resident in Ghana (n=276)		Ghanaians resident in Europe (n=313)	
	**CpG ID**	**Gene Name[1]**	**Feature[1]**	**Delta -beta value[2]**	**P. Value**	**Delta-beta value[2]**	**P. Value**
1	cg14653250	*PC*	TSS200	0.000811	6.61E-06	0.000086	8.39E-02
2	cg02338947	*FAM124B*	3'UTR	-0.006382	6.47E-09	-0.004121	9.85E-07
3	cg01573121	*DNAJC28*	5'UTR	-0.001771	3.95E-07	-0.000767	2.43E-03
4	cg12144754	*PRPS1L1*	1stExon	-0.000123	2.85E-01	-0.001927	2.75E-11
5	cg26859186	*PTPRN2*	Body	-0.001349	2.64E-04	-0.002134	1.95E-09
6	cg19712490	*CD81*	TSS200	0.000028	9.38E-01	0.004775	1.91E-10
7	cg12842013	*HOMEZ*	1stExon	0.000779	6.51E-09	0.000005	8.44E-01
8	cg01099220	*LRRC14*	3'UTR	0.000975	2.85E-04	0.001317	7.90E-04
9	cg25806492	*SRRM1*	Body	0.002884	8.72E-09	-0.000001	9.60E-01
10	cg13767940	*BTG4*	TSS200	0.003564	8.97E-08	-0.000118	7.52E-01
11	cg21010178	*PADI1*	TSS1500	-0.003397	5.68E-08	-0.000299	3.75E-01
12	cg22602019	*FAM167B*	3'UTR	0.000147	1.49E-01	0.001680	7.59E-09
13	cg13198133	Intergenic	Intergenic	-0.000445	3.40E-01	-0.005389	7.12E-10
14	cg02150674	*PHYH*	TSS200	-0.000005	9.11E-01	0.001096	1.50E-10

[1] Annotation were perfomed via IlluminaHumanMethylation450kanno.ilmn12. hg19. Homo sapiens (human) genome assembly GRCh37 (hg19) . *Hansen KD (2016) IlluminaHumanMethylation450kanno.ilmn12.hg19: Annotation for Illumina's 450k methylation arrays*. *R package version 0.6.0.*

[2] Delta β-value from linear regression model of DNA methylation against CRP adjusted for age, sex, array, plate position, alcohol consumption, smoking, BMI and proportion of immune cells. CRP levels are in mg/L.

Abbreviations:

TSS1500 – transcription start site 1500 (the region from Transcription start site (TSS) to − 1500 nucleotides upstream of TSS)

5’UTR - 5′ untranslated region (the region of an mRNA that is directly upstream from the initiation codon)

3’UTR - 3′ untranslated region (the region of an mRNA that is directly downstream from the stop codon)

TSS200 – transcription start site 200 (the region from Transcription start site (TSS) to − 200 nucleotides upstream of TSS)

	Slightly larger effect size
	Slightly smaller effect size

Supplementary Figure 3. Sensitivity analyses on location of residence (Ghanaians in Ghana vs Ghanaians in Europe.

a) Pearsons correlations between delta-Beta values in linear regression models with and without location of residence as a covariate

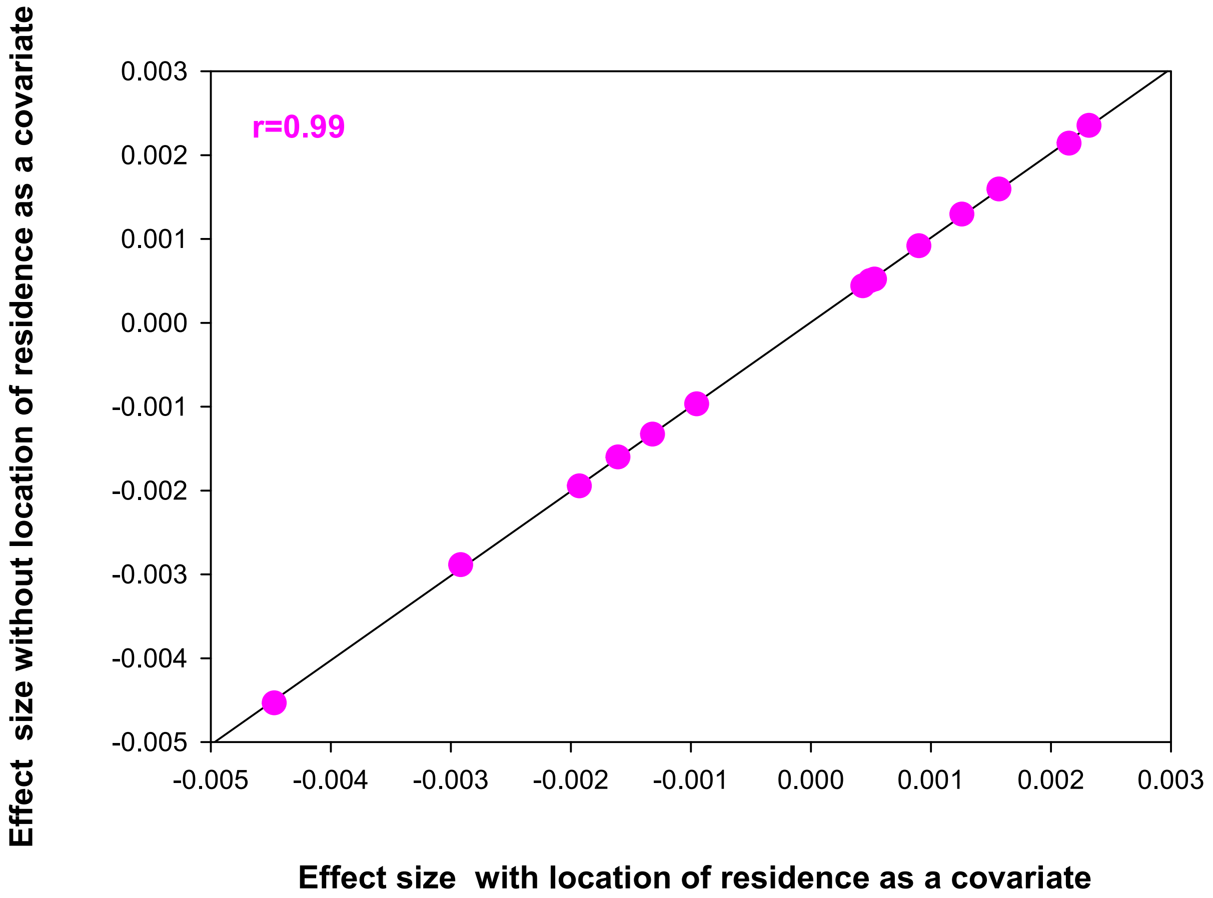

b) Pearsons correlations between P-values in linear regression models with and without location of residence as a covariate

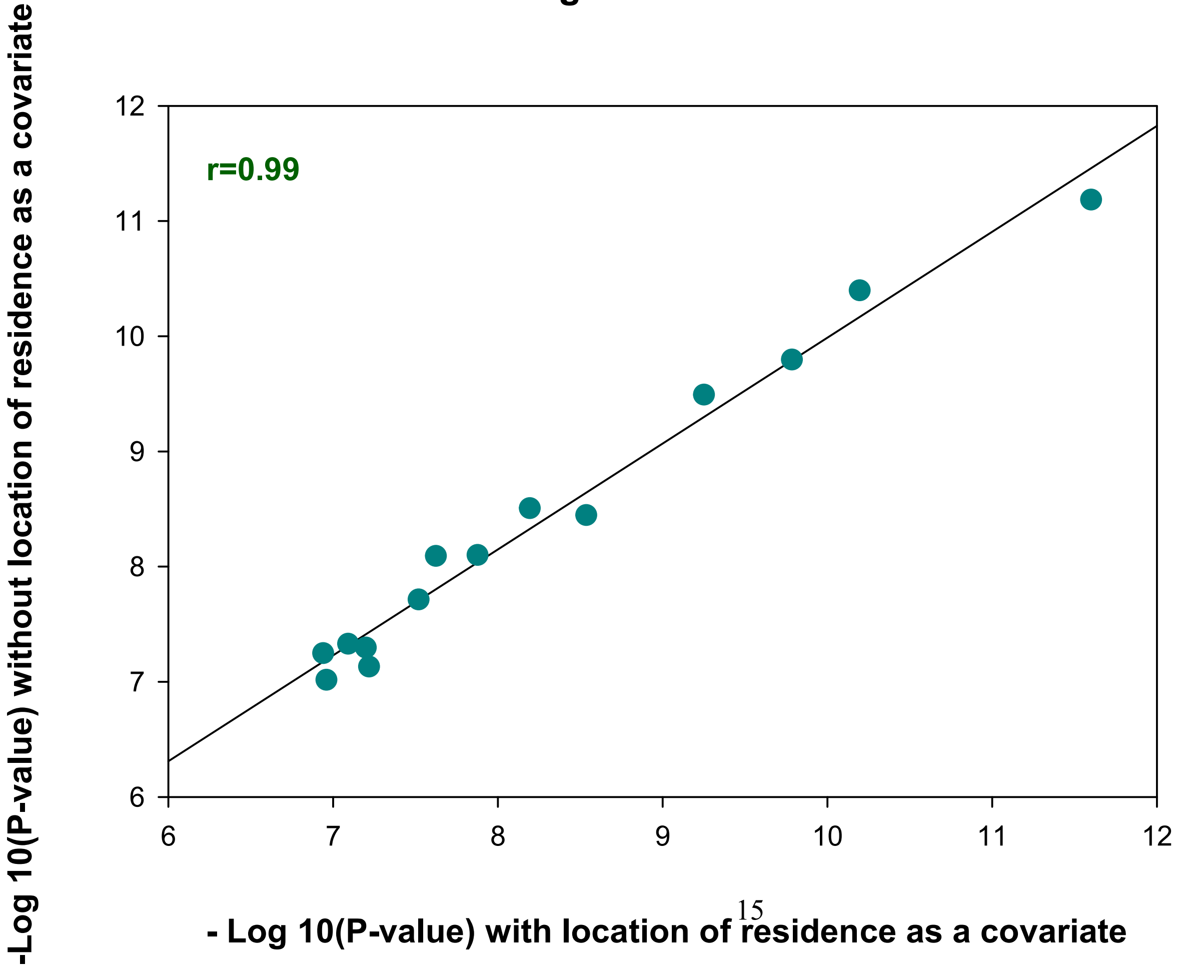

Supplementary Figure 4. Manhattan plot of DMPs associated with CRP ≤ 10 mg/L among Ghanaians. All 429,459 CpG sites are presented according to p-value in EWAS, as well as by chromosomal annotation. Red line is the demarcation line for statistically significant DMPs at p<1.1E-7.

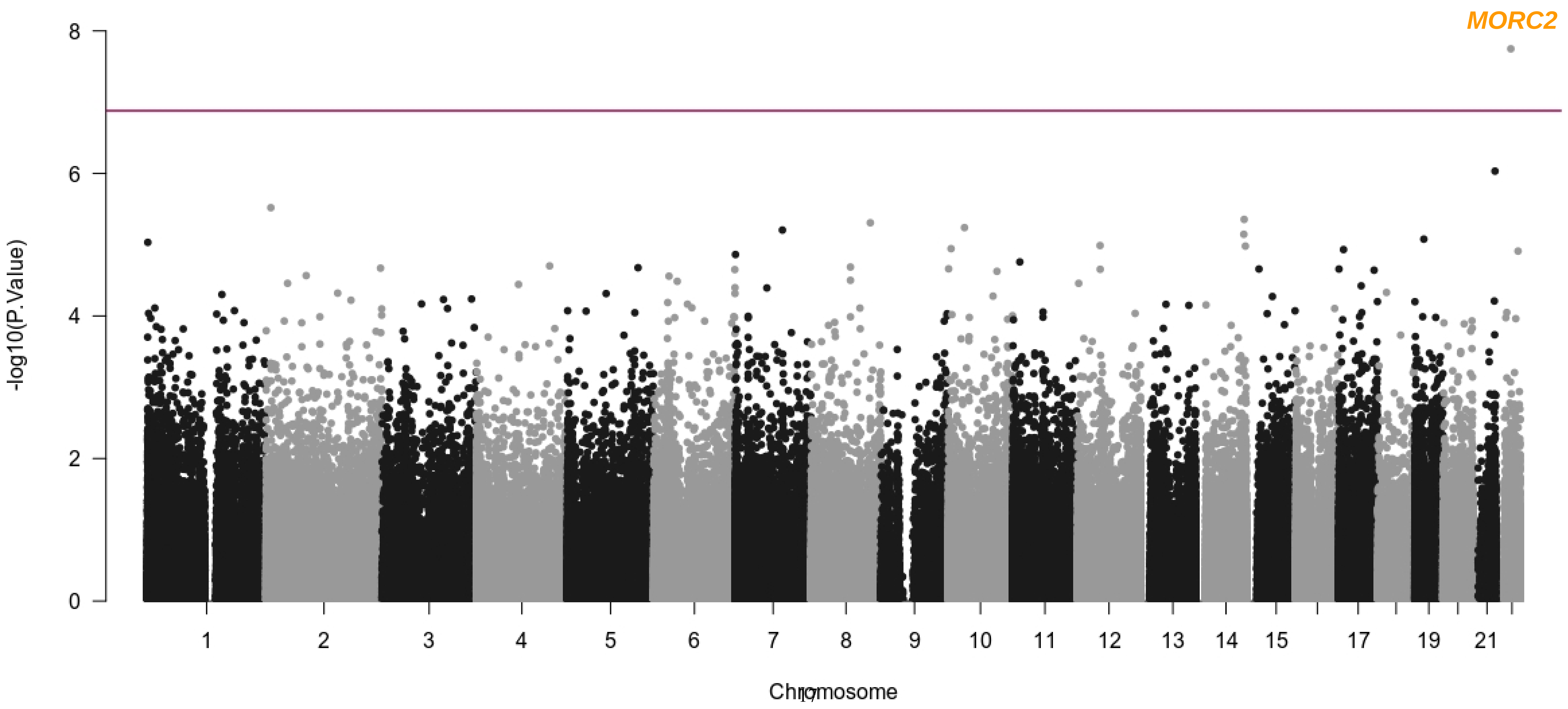
MORC2
-log10(P.Value)
8
6
4
2
0
1
2
3
4
5
6
7
8
9
10
11
12
13
14
15
17
19
21
Chromosome

Supplementary Figure 5. Inflation modelling with QQ plots for CRP levels $\leq$ 10 mg/L

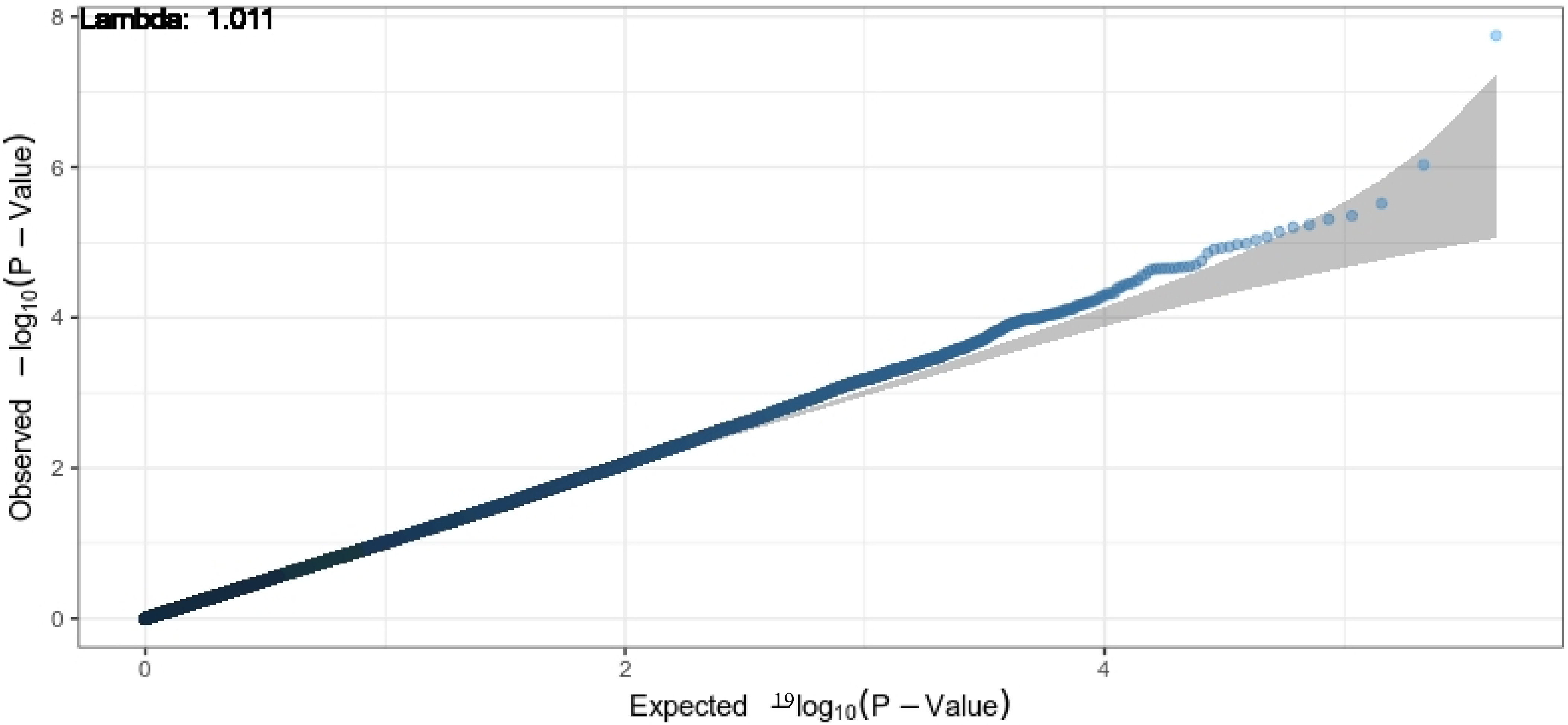

Lambda: 1.011
0
2
4
6
8
Observed −log10(P − Value)
Expected −log10(P − Value)

Supplementary Table 4. Differentially methylated position (DMP) associated with CRP at levels ≤ 10 mg/L

a) ***Gene annotation and Effect size:***

No	CpG ID	Chromosome	Position[1]	Gene Name[1]	Feature[1]	Relation to Island[1]	Delta β value	P. Value	FDR[6]
1	cg02551882	Chr22	31365455	*MORC2*	TSS1500	Island	0.00041	1.78e-08	0.0076

[1] Annotation were perfomed via IlluminaHumanMethylation450kanno.ilmn12.hg19. Homo sapiens (human) genome assembly GRCh37 (hg19) . *Hansen KD (2016) IlluminaHumanMethylation450kanno.ilmn12.hg19: Annotation for Illumina's 450k methylation arrays. R package version 0.6.0.*

[2] Delta β-value of DNA methylation against each unit change in CRP (mg/L) derived from linear regression model adjusted for age, sex, array, plate position, alcohol consumption, smoking, BMI and proportion of immune cells.

TSS1500 – transcription start site 1500 (the region from Transcription start site (TSS) to − 1500 nucleotides upstream of TSS).

b) Relationship between DNA methylation and gene expression as reported in the IMETHYL database:

CpG ID	Nearest Gene[1]	Gene Feature[1]	Methylation level [2]	Methylation average[2] % (SD)	FPKM[2,3] average (SD)
cg02551882	*MORC2*	TSS1500	Low	2.33(2.61)	1.40(0.06)

[1] Annotation were perfomed via IlluminaHumanMethylation450kanno.ilmn12.hg19. Homo sapiens (human) genome assembly GRCh37 (hg19). *Hansen KD (2016) IlluminaHumanMethylation450kanno.ilmn12.hg19: Annotation for Illumina's 450k methylation arrays. R package version 0.6.0.*

[2] Methylation level according to iMETHYL database (low, medium, high). IMETHYL provides whole-DNA methylation (~24 million autosomal CpG sites), whole-genome (~9 million single-nucleotide variants), and whole-transcriptome (>14 000 genes) data for $CD4^+$ T-lymphocytes, monocytes, and neutrophils collected from approximately 100 subjects. *Komaki, S., Shiwa, Y., Furukawa, R. et al. iMETHYL: an integrative database of human DNA methylation, gene expression, and genomic variation. Hum Genome Var 5, 18008 (2018).*

[3] FPKM= Fragments Per Kilobase of transcript per Million mapped reads

c) Associations of DMP with estimated risk of cardiovascular diseases (ASCVD) among Ghanaians.

CpG ID	Nearest Gene[1]	Gene Feature[1]	Beta coefficient[2]	P-value
cg02551882	*MORC2*	TSS1500	0.0006	0.2508

[1] Annotation were perfomed via IlluminaHumanMethylation450kanno.ilmn12. hg19. Homo sapiens (human) genome assembly GRCh37 (hg19). *Hansen KD (2016) IlluminaHumanMethylation450kanno.ilmn12.hg19: Annotation for Illumina's 450k methylation arrays. R package version 0.6.0.*

[2] β-coefficient from linear regression model of DNA methylation in CRP (outcome) against estimated risk of cardiovascular diseases adjusted for alcohol consumption, BMI and proportion of immune cells. N=472

ASCVD risk= American College of Cardiology/American Heart Association atherosclerotic cardiovascular disease (ACC/AHA ASCVD) risk score as previously applied in the RODAM study. The risk score is used among persons aged 40–79 years, without prior history of CVD, using an algorithm that combines age, sex, use of antihypertensive medication, systolic blood pressure, presence of T2D, total cholesterol, HDL cholesterol and smoking status. A score of >7.5% is considered to be an elevated risk of developing a CVD in the next 10 years.

d) ***Gene function in previous Epigenome wide association studies (EWAS) as reported in the EWAS catalog***:

Gene Name	Gene Function	Associated Phenotypes	Phenotypes linked to inflammation or CVD
MORC2	This gene encodes a protein known to regulate the condensation of heterochromatin in response to DNA damage and play a role in repressing transcription. The protein has been found to regulate the activity of ATP citrate lyase via specific interaction with this enzyme in the cytosol of lipogenic breast cancer cells. The protein also plays a role in lipogenesis and adipocyte differentiation.	• Smoking<br>• Primary Sjogrens syndrome<br>• Papuan ancestry<br>• Gestational age	• Smoking<br>• Primary Sjogrens syndrome

e) Gene function in previous genome wide association studies (GWAS) as reported in the GWAS catalog or Gene Hancer:

Gene Name	Gene Function	Associated Phenotypes	Phenotypes linked to inflammation or CVD
MORC2	This gene encodes a protein known to regulate the condensation of heterochromatin in response to DNA damage and play a role in repressing transcription. The protein has been found to regulate the activity of ATP citrate lyase via specific interaction with this enzyme in the cytosol of lipogenic breast cancer cells. The protein also plays a role in lipogenesis and adipocyte differentiation	• Heel bone mineral density<br>• BMI-adjusted waist circumference<br>• Vital capacity<br>• Blood protein measurement<br>• Type ii diabetes mellitus<br>• Moyamoya disease<br>• Fev/fec ratio<br>• Eosinophil count<br>• Acute myeloid leukemia<br>• Intelligence<br>• Isoleucine measurement<br>• Diabetic nephropathy<br>• Skin pigmentation<br>• Parental longevity<br>• Coronary artery calcification<br>• Reaction time measurement	• BMI-adjusted waist circumference<br>• Type II diabetes mellitus<br>• Eosinophil count<br>• Diabetic nephropathy<br>• Coronary artery calcification

Supplementary Table 5. Pathway analysis of genome wide significant DMPs in the GO (Gene Ontology) database.

PATHWAY	ONTOLOGY	TERM	N	DE	P.DE	FDR
GO:0019606	BP	2-oxobutyrate catabolic process	1	1	0.00063	1
GO:0048244	MF	phytanoyl-CoA dioxygenase activity	1	1	0.00063	1
GO:0043126	BP	regulation of 1-phosphatidylinositol 4-kinase activity	1	1	0.00093	1
GO:0043128	BP	positive regulation of 1-phosphatidylinositol 4-kinase activity	1	1	0.00093	1
GO:1905676	BP	positive regulation of adaptive immune memory response	1	1	0.00093	1
GO:0004736	MF	pyruvate carboxylase activity	1	1	0.00101	1
GO:0046361	BP	2-oxobutyrate metabolic process	2	1	0.00151	1
GO:0090716	BP	adaptive immune memory response	2	1	0.00169	1
GO:1905674	BP	regulation of adaptive immune memory response	2	1	0.00169	1
GO:0048213	BP	Golgi vesicle prefusion complex stabilization	2	1	0.00185	1
GO:0019072	BP	viral genome packaging	2	1	0.00216	1
GO:0019074	BP	viral RNA genome packaging	2	1	0.00216	1
GO:0097089	BP	methyl-branched fatty acid metabolic process	3	1	0.00229	1
GO:2001190	BP	positive regulation of T cell activation via T cell receptor contact with antigen bound to MHC molecule on antigen presenting cell	3	1	0.00240	1
GO:0046813	BP	receptor-mediated virion attachment to host cell	3	1	0.00252	1
GO:0035783	BP	CD4-positive, alpha-beta T cell stimulation	3	1	0.00257	1
GO:0002189	CC	ribose phosphate diphosphokinase complex	3	1	0.00277	1

GO:0004749	MF	ribose phosphate diphosphokinase activity	3	1	0.00277	1
GO:1904352	BP	positive regulation of protein catabolic process in the vacuole	4	1	0.00285	1
GO:0044789	BP	modulation by host of viral release from host cell	4	1	0.00309	1

N= number of genes in the GO term

DE=number of genes that are differentially methylated

P.DE=p-value for over-representation of the GO or KEGG term

FDR=False discovery rate

Supplementary Table 6. Pathway analysis of genome wide significant DMPs in the KEGG (Kyoto Encyclopedia of Genes and Genomes) database.

Pathways	Description	N	DE	P.DE	FDR
path:hsa01230	Biosynthesis of amino acids	64	2	0.001221	0.417554
path:hsa01200	Carbon metabolism	106	2	0.003321	0.5679
path:hsa00030	Pentose phosphate pathway	24	1	0.01898	1
path:hsa00020	Citrate cycle (TCA cycle)	28	1	0.022738	1
path:hsa04940	Type I diabetes mellitus	41	1	0.028876	1
path:hsa00620	Pyruvate metabolism	46	1	0.034683	1
path:hsa05144	Malaria	48	1	0.036171	1
path:hsa04662	B cell receptor signaling pathway	77	1	0.057338	1
path:hsa04146	Peroxisome	80	1	0.062692	1
path:hsa03015	mRNA surveillance pathway	84	1	0.066832	1
path:hsa00230	Purine metabolism	123	1	0.087058	1
path:hsa05160	Hepatitis C	141	1	0.10139	1
path:hsa03013	RNA transport	148	1	0.116213	1
path:hsa01100	Metabolic pathways	1403	2	0.300242	1
path:hsa00010	Glycolysis / Gluconeogenesis	63	0	1	1
path:hsa00040	Pentose and glucuronate interconversions	30	0	1	1
path:hsa00051	Fructose and mannose metabolism	32	0	1	1
path:hsa00052	Galactose metabolism	28	0	1	1
path:hsa00053	Ascorbate metabolism	26	0	1	1
path:hsa00061	Fatty acid biosynthesis	16	0	1	1

N= number of genes in the KEGG term

DE= number of genes that are differentially methylated

P.DE=p-value for over-representation of the GO or KEGG term

FDR=False discovery rate

Supplementary Table 7. Gene function and associated phenotypes in EWAS catalog

Gene Name	Gene Function	Associated Phenotypes	Phenotypes linked to inflammation.
**Top genes identified in our EWAS**			
PC	This gene encodes pyruvate carboxylase, which requires biotin and ATP to catalyse the carboxylation of pyruvate to oxaloacetate. The active enzyme is a homotetramer arranged in a tetrahedron which is located exclusively in the mitochondrial matrix. Pyruvate carboxylase is involved in gluconeogenesis, lipogenesis, insulin secretion and synthesis of the neurotransmitter glutamate.	• Clear cell renal carcinoma<br>• Fetal vs adult liver<br>• HIV infection<br>• Gestational age<br>• Rheumatoid arthritis<br>• Primary Sjogrens syndrome<br>• Mortality<br>• Long-term prenatal exposure to paracetamol in children with ADHD versus controls<br>• Sex<br>• Current versus never smoking.<br>• Maternal smoking in pregnancy<br>• Multiple sclerosis<br>• Air pollution exposure<br>• Proinsulin<br>• Psoriasis<br>• Acetoacetate	• HIV infection<br>• Rheumatoid arthritis<br>• Primary Sjogrens syndrome<br>• Multiple sclerosis<br>• Air pollution exposure<br>• Proinsulin<br>• Psoriasis
FAM124B	This gene is an interacting partner of a CHD7 and CHD8 containing complex which has been linked to autism spectrum disorders.	• Age 4 vs age 0<br>• Fetal vs adult liver<br>• Smoking<br>• Sex<br>• Gestational age<br>• Rheumatoid arthritis<br>• Pancreatic ductal adenocarcinoma<br>• HIV infection	• Rheumatoid arthritis<br>• HIV infection<br>• Smoking

DNAJC28	This gene encodes a member of the DnaJ heat shock protein family. The encoded protein, which contains a conserved N-terminal DnaJ domain, is thought to play a role in protein folding or act as a molecular chaperone protein	• Rheumatoid arthritis<br>• Age<br>• Gestational age<br>• HIV infection	• HIV infection<br>• Rheumatoid arthritis
PRPS1L1	This intronless gene is specifically expressed in the testis and encodes a protein that is highly homologous to the two subunits of phosphoribosylpyrophosphate synthetase encoded by human X-linked genes, PRPS1 and PRPS2. These enzymes convert pyrimidine, purine, or pyridine bases to the corresponding ribonucleoside monophosphates.	• Primary Sjogrens syndrome<br>• HIV infection<br>• Rheumatoid arthritis	• Primary Sjogrens syndrome<br>• HIV infection<br>• Rheumatoid arthritis
PTPRN2	This gene encodes a protein with sequence similarity to receptor-like protein tyrosine phosphatases. This protein has been identified as an autoantigen in insulin-dependent diabetes mellitus.	• Clear cell renal carcinoma<br>• Age 4 vs age 0<br>• Fetal vs adult liver<br>• Sex<br>• Pancreatic ductal adenocarcinoma<br>• Primary Sjogrens syndrome<br>• Rheumatoid arthritis<br>• Crohn's disease<br>• HIV infection<br>• Gestational age<br>• Sex<br>• Inflammatory bowel disease<br>• Aging<br>• Papuan ancestry<br>• Chronic kidney disease<br>• Ulcerative colitis<br>• Fetal intolerance of labor<br>• Leg bone mineral density<br>• Ulcerative colitis	• Primary Sjogrens syndrome<br>• Rheumatoid arthritis<br>• Crohn's disease<br>• HIV infection<br>• Inflammatory bowel disease<br>• Ulcerative colitis<br>• Cholesterol esters to total lipids ratio in IDL<br>• N-terminal pro-B-type natriuretic peptide<br>• Hypertensive disorders of pregnancy<br>• Total fat free mass<br>• Total lean mass

		• Cholesterol esters to total lipids ratio in IDL<br>• Arsenic exposure<br>• Total body naevus count<br>• Albumin<br>• Alpha neck angle (hip measurement)<br>• Spine bone mineral density<br>• Mortality<br>• N-terminal pro-B-type natriuretic peptide<br>• Progressive supranuclear palsy<br>• Long-term prenatal exposure to paracetamol in children with ADHD versus controls<br>• Total fat free mass<br>• Total lean mass<br>• Hypertensive disorders of pregnancy<br>• Aircraft noise pollution<br>• Alanine<br>• Schizophrenia	
CD81	The protein encoded by this gene is a member of the transmembrane 4 superfamily, also known as the tetraspanin family. Most of these members are cell-surface proteins that are characterized by the presence of four hydrophobic domains. The proteins mediate signal transduction events that play a role in the regulation of cell development, activation, growth, and motility. Among its related pathways are IL-2 Pathway.	• Gestational age<br>• Smoking pack-years<br>• HIV infection<br>• Maternal urinary arsenic level<br>• Papuan ancestry<br>• Alcohol consumption per day<br>• Fasting glucose<br>• HIV infection<br>• Inflammatory bowel disease<br>• Cognitive abilities: digit test<br>• Alzheimer's disease<br>• Schizophrenia<br>• Asthma<br>• Sex<br>• Long-term prenatal exposure to paracetamol in children with ADHD versus control<br>• Alcohol consumption per day<br>• Age	• Smoking pack-years<br>• HIV infection<br>• Alcohol consumption per day<br>• Fasting glucose<br>• HIV infection<br>• Inflammatory bowel disease<br>• Asthma

HOMEZ	This gene is a Homeobox gene that may function as a transcriptional regulator	• HIV infection<br>• Sex<br>• Fetal alcohol spectrum disorder	• HIV infection
LRRC14	This gene encodes a leucine-rich repeat-containing protein. It Negatively regulates Toll-like receptor-mediated NF-kappa-B signaling by disrupting IKK core complex formation through interaction with IKBKB	• Clear cell renal carcinoma<br>• Rheumatoid arthritis<br>• HIV infection<br>• Maternal Psychopathology<br>• Fetal alcohol spectrum disorder<br>• Mean diameter for VLDL particles	• Rheumatoid arthritis<br>• HIV infection<br>• Mean diameter for VLDL particles
SRRM1	Part of pre- and post-splicing multiprotein mRNP complexes. Involved in numerous pre-mRNA processing events. Promotes constitutive and exonic splicing enhancer (ESE)-dependent splicing activation by bridging together sequence-specific (SR family proteins, SFRS4, SFRS5 and TRA2B/SFRS10) and basal snRNP (SNRP70 and SNRPA1) factors of the spliceosome.	• Age 4 vs age 0<br>• Fetal vs adult liver<br>• Rheumatoid arthritis<br>• HIV infection	• Fetal vs adult liver<br>• Rheumatoid arthritis<br>• HIV infection
BTG4	The protein encoded by this gene is a member of the BTG/Tob family. This family has structurally related proteins that appear to have antiproliferative properties. This encoded protein can induce G1 arrest in the cell cycle.	• Pre-eclampsia<br>• Rheumatoid arthritis	• Pre-eclampsia<br>• Rheumatoid arthritis
PADI1	This gene encodes a member of the peptidyl arginine deiminase family of enzymes, which catalyze the post-translational deamination of proteins by converting arginine residues into citrullines in the presence of calcium ions. The family members have distinct substrate specificities and tissue-specific expression patterns.	• Age 4 vs age 0<br>• Gestational age<br>• Aging<br>• Rheumatoid arthritis<br>• Maternal smoking in pregnancy<br>• HIV infection<br>• Papuan ancestry<br>• Optimal NICU Network Neurobehavioral Scale Profile	• Rheumatoid arthritis<br>• Maternal smoking in pregnancy<br>• HIV infection

FAM167B	Micro arrays show that FAM167B has varied expression in reactions to cancers, but no information regarding the exact function of FAM167B can be drawn from these micro arrays. FAM167B has a higher expression in the skin, B-cells, and spleen, but the same low expression in all other cell types	• Fetal vs adult liver<br>• Gestational age<br>• Rheumatoid arthritis<br>• Age<br>• Hypertensive disorders of pregnancy	• Rheumatoid arthritis<br>• Hypertensive disorders of pregnancy
PHYH	This gene is a member of the Antp homeobox family and encodes a protein with a homeobox DNA-binding domain. This nuclear protein functions as a sequence-specific transcription factor that is involved in differentiation and limb development	• Primary Sjogrens syndrome<br>• HIV infection<br>• Rheumatoid arthritis<br>• Age<br>• Schizophrenia<br>• Gestational age<br>• Papuan ancestry	• Primary Sjogrens syndrome<br>• HIV infection<br>• Rheumatoid arthritis

Supplementary Table 8. Gene function and associated phenotypes in GWAS catalog and GeneHancer database.

Gene Name	Gene Function	Associated Phenotypes	Phenotypes linked to inflammation.
**Top genes identified in our EWAS**			
PC	This gene encodes pyruvate carboxylase, which requires biotin and ATP to catalyse the carboxylation of pyruvate to oxaloacetate. The active enzyme is a homotetramer arranged in a tetrahedron which is located exclusively in the mitochondrial matrix. Pyruvate carboxylase is involved in gluconeogenesis, lipogenesis, insulin secretion and synthesis of the neurotransmitter glutamate.	• Morningness<br>• Reaction time<br>• Familial squamous cell lung carcinoma<br>• Bipolar disorder<br>• Waist circumference adjusted for body mass index.<br>• Urate levels<br>• Educational attainment (years of education)<br>• Lung function (FVC)<br>• Hair color<br>• HIV-1 susceptibility<br>• Total PHF-tau (SNP x SNP interaction)	• Urate levels<br>• HIV-1 susceptibility<br>• Waist circumference adjusted for body mass index.
FAM124B	This gene is a potential interacting partner of a CHD7 and CHD8 containing complex.	• Platelet-derived growth factor BB levels<br>• Hematuria<br>• Family history of lung cancer, squamous cell lung carcinoma<br>• Monokine induced by gamma interferon measurement.<br>• Fear of minor pain measurement	• Platelet-derived growth factor BB levels<br>• Monokine induced by gamma interferon measurement.
DNAJC28	This gene encodes a member of the DnaJ heat shock protein family. The encoded protein, which contains a conserved N-terminal DnaJ domain, is thought to play a role in protein folding or act as a molecular chaperone protein	• Ankylosing<br>• Spondylitis,<br>• Psoriasis<br>• Ulcerative colitis<br>• Crohn's disease<br>• Sclerosing cholangitis	• Ankylosing<br>• Spondylitis,<br>• Psoriasis<br>• Ulcerative colitis<br>• Crohn's disease<br>• Sclerosing cholangitis

PRPS1L1	This intronless gene is specifically expressed in the testis and encodes a protein that is highly homologous to the two subunits of phosphoribosylpyrophosphate synthetase encoded by human X-linked genes, PRPS1 and PRPS2. These enzymes convert pyrimidine, purine, or pyridine bases to the corresponding ribonucleoside monophosphates.	• Susceptibility to measles measurement<br>• Fibrinogen measurement	• Susceptibility to measles measurement<br>• Fibrinogen measurement
PTPRN2	This gene encodes a protein with sequence similarity to receptor-like protein tyrosine phosphatases. This protein has been identified as an autoantigen in insulin-dependent diabetes mellitus.	• Smoking initiation (ever regular vs never regular) (MTAG), Smoking status<br>• Mathematical ability<br>• Gut microbiome measurement<br>• Intelligence<br>• Adolescent idiopathic scoliosis<br>• Body mass index<br>• Cognitive function measurement<br>• Risk-taking behavior<br>• Mental or behavioral disorder<br>• Response to drug<br>• Energy intake<br>• Pathological myopia<br>• Diastolic blood pressure<br>• Susceptibility to scarlet fever measurement<br>• Risky sexual behavior measurement<br>• Atopic eczema, psoriasis<br>• Cholesteryl ester 14:0 measurement<br>• Calcium measurement<br>• Colorectal cancer<br>• Coronary artery calcification,<br>• Type ii diabetes mellitus<br>• Triglyceride measurement<br>• Non-lobar intracerebral hemorrhage<br>• Reaction time measurement<br>• Ovarian carcinoma<br>• Intelligence, schizophrenia, self-reported educational attainment	• Smoking initiation<br>• Body mass index<br>• Energy intake<br>• Diastolic blood pressure<br>• Atopic eczema, psoriasis<br>• Cholesteryl ester 14:0 measurement<br>• Coronary artery calcification,<br>• Type ii diabetes mellitus<br>• Triglyceride measurement<br>• Carotid plaque build

		• Self-reported educational attainment, cognitive function measurement<br>• Self-reported educational attainment<br>• Age at menarche<br>• Carotid plaque build<br>• Breast carcinoma, childhood cancer, response to radiation<br>• Childhood cancer, breast carcinoma<br>• Chronic kidney disease<br>• Platinum measurement, response to cisplatin<br>• Thyroid peroxidase antibody measurement<br>• Amyloid-beta measurement	
CD81	The protein encoded by this gene is a member of the transmembrane 4 superfamily, also known as the tetraspanin family. Most of these members are cell-surface proteins that are characterized by the presence of four hydrophobic domains. The proteins mediate signal transduction events that play a role in the regulation of cell development, activation, growth, and motility. Among its related pathways are IL-2 Pathway.	• Type 2 diabetes<br>• Bacteriemia<br>• Systemic scleroderma<br>• Monocyte count<br>• Breast carcinoma	• Type 2 diabetes<br>• Systemic scleroderma<br>• Monocyte count
HOMEZ	This gene is a Homeobox gene that may function as a transcriptional regulator	• Pulse pressure<br>• High light scatter reticulocyte count, Reticulocyte count<br>• Systolic blood pressure<br>• Height<br>• Waist-to-hip ratio adjusted for BMI.<br>• Serum alkaline phosphatase levels<br>• High light scatter reticulocyte percentage of red cells<br>• Mean platelet volume<br>• Platelet count<br>• White blood cell count	• Pulse pressure<br>• Systolic blood pressure<br>• White blood cell count

LRRC14	This gene encodes a leucine-rich repeat-containing protein. It Negatively regulates Toll-like receptor-mediated NF-kappa-B signaling by disrupting IKK core complex formation through interaction with IKBKB	• Intelligence, intelligence (mtag)<br>• Educational attainment<br>• Cognitive ability (mtag)<br>• Cognitive performance<br>• Cognitive aspects of educational attainment<br>• Household income (mtag)<br>• Eosinophil count<br>• Low density lipoprotein cholesterol measurement<br>• Eosinophil percentage of leukocytes<br>• Neutrophil percentage of granulocytes<br>• Total cholesterol measurement<br>• Asthma<br>• Fibrinogen measurement<br>• Smoking status measurement<br>• Systolic blood pressure<br>• Lean body mass<br>• Glucagon-like peptide-1 measurement, glucose tolerance test	• Eosinophil count<br>• Low density lipoprotein cholesterol measurement<br>• Eosinophil percentage of leukocytes<br>• Neutrophil percentage of granulocytes<br>• Total cholesterol measurement<br>• Asthma<br>• Fibrinogen measurement<br>• Smoking status measurement<br>• Systolic blood pressure<br>• Lean body mass<br>• Glucagon-like peptide-1 measurement, glucose tolerance test
SRRM1	Part of pre- and post-splicing multiprotein mRNP complexes. Involved in numerous pre-mRNA processing events. Promotes constitutive and exonic splicing enhancer (ESE)-dependent splicing activation by bridging together sequence-specific (SR family proteins, SFRS4, SFRS5 and TRA2B/SFRS10) and basal snRNP (SNRP70 and SNRPA1) factors of the spliceosome.	• Red blood cell count<br>• Male-pattern baldness<br>• HDL cholesterol levels<br>• Apolipoprotein A1 levels<br>• Diastolic blood pressure<br>• Adult body size<br>• Red blood cell count<br>• Hematocrit<br>• Hemoglobin<br>• Plateletcrit<br>• Platelet count<br>• Cardiovascular disease<br>• Systolic blood pressure	• HDL cholesterol levels<br>• Apolipoprotein A1 levels<br>• Diastolic blood pressure<br>• Cardiovascular disease<br>• Systolic blood pressure
BTG4	The protein encoded by this gene is a member of the BTG/Tob family. This family has structurally related proteins that appear to have antiproliferative properties. This encoded protein can induce G1 arrest in the cell cycle.	• Interleukin-4 levels<br>• Allergy or asthma<br>• Waist-to-hip-ratio	• Interleukin-4 levels<br>• Allergy or asthma<br>• Waist-to-hip ratio

PADI1	This gene encodes a member of the peptidyl arginine deiminase family of enzymes, which catalyze the post-translational deamination of proteins by converting arginine residues into citrullines in the presence of calcium ions. The family members have distinct substrate specificities and tissue-specific expression patterns.	• Serum 25-Hydroxyvitamin D levels<br>• Phosphatidylcholine<br>• IGA glomerulonephritis<br>• Diet	• Serum 25-Hydroxyvitamin D levels<br>• IGA glomerulonephritis<br>• Diet
FAM167B	Micro arrays show that FAM167B has varied expression in reactions to cancers, but no information regarding the exact function of FAM167B can be drawn from these micro arrays. FAM167B has a higher expression in the skin, B-cells, and spleen, but the same low expression in all other cell types	• None	• None
PHYH	This gene is a member of the Antp homeobox family and encodes a protein with a homeobox DNA-binding domain. This nuclear protein functions as a sequence-specific transcription factor that is involved in differentiation and limb development	• PHF-tau measurement<br>• Blood protein measurement<br>• Allergy<br>• Reading and spelling ability<br>• Kynurenic acid measurement	• Allergy<br>• Kynurenic acid

Supplementary Figure 6. Flow chart of participation

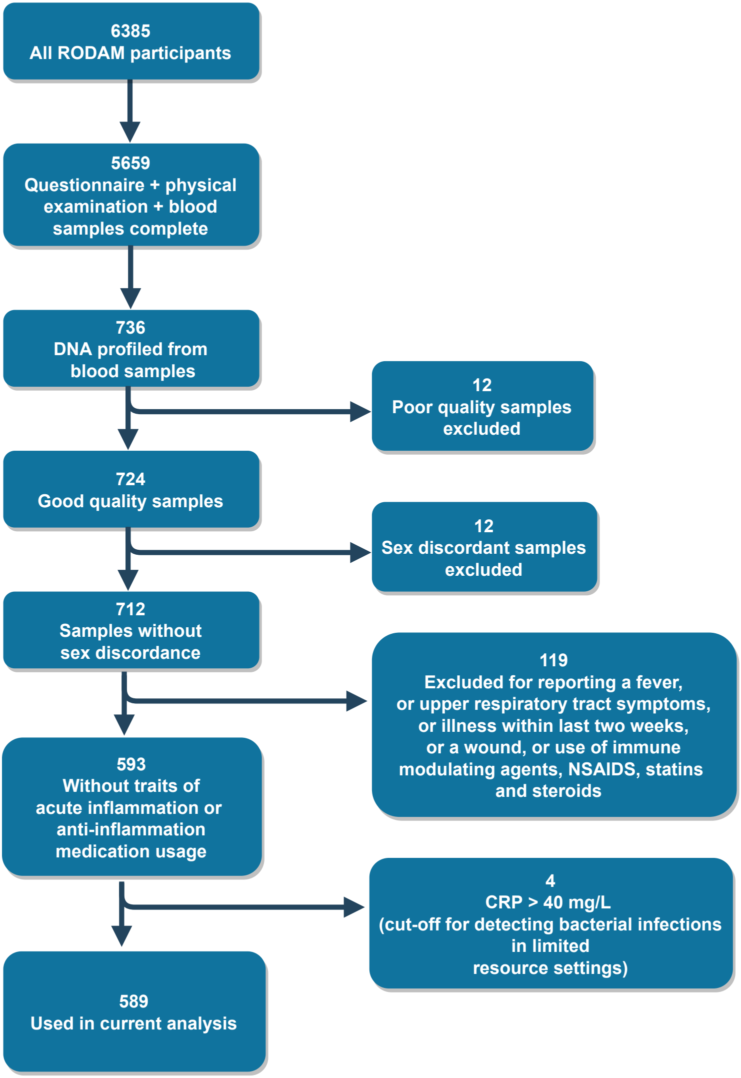
6385
All RODAM participants
5659
Questionnaire + physical examination + blood samples complete
736
DNA profiled from blood samples
12
Poor quality samples excluded
724
Good quality samples
12
Sex discordant samples excluded
712
Samples without sex discordance
119
Excluded for reporting a fever, or upper respiratory tract symptoms, or illness within last two weeks, or a wound, or use of immune modulating agents, NSAIDS, statins and steroids
593
Without traits of acute inflammation or anti-inflammation medication usage
4
CRP > 40 mg/L
(cut-off for detecting bacterial infections in limited resource settings)
589
Used in current analysis

Supplementary Figure 7. Distribution of CRP in the final sample

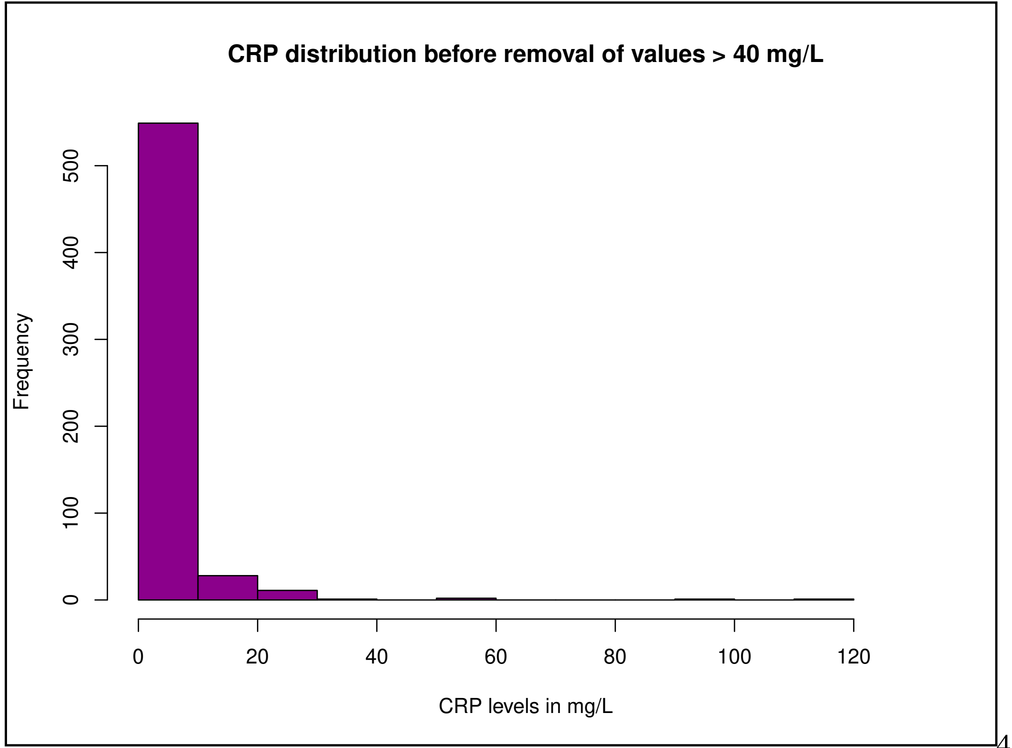
CRP distribution before removal of values > 40 mg/L
Frequency
0
100
200
300
400
500
0
20
40
60
80
100
120
CRP levels in mg/L

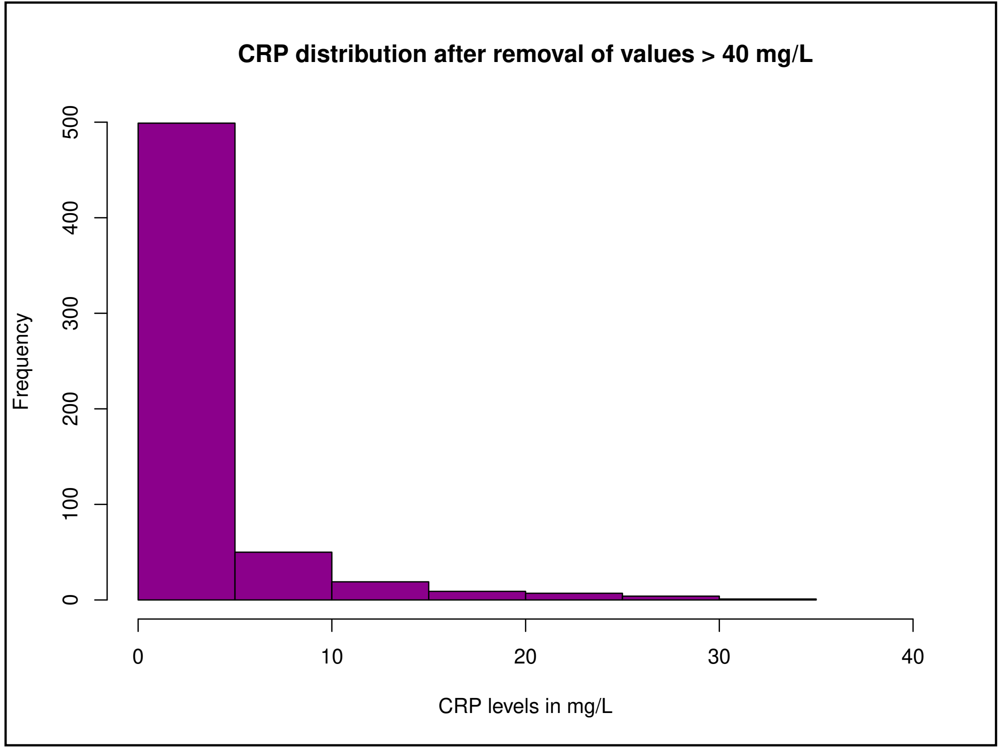
CRP distribution after removal of values > 40 mg/L
Frequency
0
100
200
300
400
500
0
10
20
30
40
CRP levels in mg/L

Supplementary Figure 8. Inflation modelling with QQ plots in the main analysis

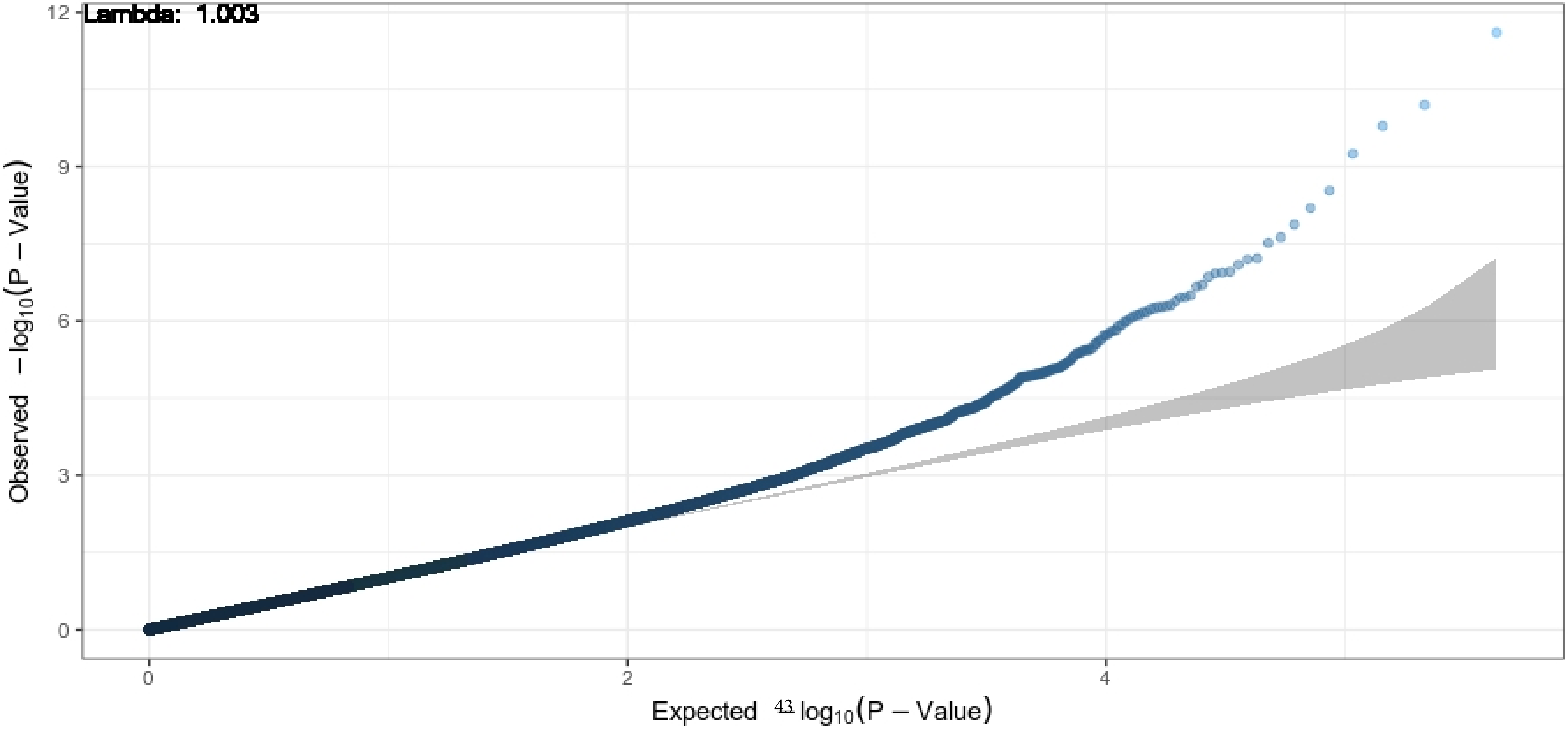

Lambda: 1.003
12
9
6
3
0
0
2
4
Observed −log10(P − Value)
Expected 43 log10(P − Value)
