# Supplementary material for: Genome-wide DNA methylation analysis on C-reactive protein among Ghanaians suggests molecular links to the emerging risk of cardiovascular diseases
Source: NPJ Genom Med. 2021 Jun 11;6:46. doi: 10.1038/s41525-021-00213-9 (PMC8196035; doi:10.1038/s41525-021-00213-9)
Supplement: Supplementary file 2 — Reporting Summary [file 41525_2021_213_MOESM2_ESM.pdf]

nature research

Corresponding author(s): Felix P Chilunga

Last updated by author(s): 20-05-2021

Reporting Summary

Nature Research wishes to improve the reproducibility of the work that we publish. This form provides structure for consistency and transparency in reporting. For further information on Nature Research policies, see our Editorial Policies and the Editorial Policy Checklist.

Statistics

For all statistical analyses, confirm that the following items are present in the figure legend, table legend, main text, or Methods section.

n/a	Confirmed	
☐	☒	The exact sample size (*n*) for each experimental group/condition, given as a discrete number and unit of measurement
☐	☒	A statement on whether measurements were taken from distinct samples or whether the same sample was measured repeatedly
☐	☒	The statistical test(s) used AND whether they are one- or two-sided<br>*Only common tests should be described solely by name; describe more complex techniques in the Methods section.*
☐	☒	A description of all covariates tested
☐	☒	A description of any assumptions or corrections, such as tests of normality and adjustment for multiple comparisons
☐	☒	A full description of the statistical parameters including central tendency (e.g. means) or other basic estimates (e.g. regression coefficient) AND variation (e.g. standard deviation) or associated estimates of uncertainty (e.g. confidence intervals)
☐	☒	For null hypothesis testing, the test statistic (e.g. *F*, *t*, *r*) with confidence intervals, effect sizes, degrees of freedom and *P* value noted<br>*Give P values as exact values whenever suitable.*
☒	☐	For Bayesian analysis, information on the choice of priors and Markov chain Monte Carlo settings
☒	☐	For hierarchical and complex designs, identification of the appropriate level for tests and full reporting of outcomes
☐	☒	Estimates of effect sizes (e.g. Cohen's *d*, Pearson's *r*), indicating how they were calculated

Our web collection on statistics for biologists contains articles on many of the points above.

Software and code

Policy information about availability of computer code

Data collection	The data collected and entered through the LimeSurvey and Oracle Clinical. LimeSurvey is a free and open source on-line statistical survey web app written in PHP based on a MySQL, SQLite, PostgreSQL or MSSQL database, distributed under the GNU General Public License. Oracle Clinical is a clinical data management system that includes subsystems for study design, randomization, data entry, batch data load, lab ranges, custom validation and derivation procedures, discrepancy management, and data extract.
Data analysis	Data analysis was conducted in R. R is a programming language and free software environment for statistical computing and graphics supported by the R Foundation for Statistical Computing.

For manuscripts utilizing custom algorithms or software that are central to the research but not yet described in published literature, software must be made available to editors and reviewers. We strongly encourage code deposition in a community repository (e.g. GitHub). See the Nature Research guidelines for submitting code & software for further information.

Data

Policy information about availability of data

All manuscripts must include a data availability statement. This statement should provide the following information, where applicable:

- Accession codes, unique identifiers, or web links for publicly available datasets
- A list of figures that have associated raw data
- A description of any restrictions on data availability

Individual participant data from the RODAM study used in the current analyses has been deposited to the European Genome-Phenome Archive (https://ega-archive.org/) in a deidentified or anonymised format. Accession number is EGAS00001005162. Data will be shared with researchers submitting a research proposal and requesting access to data. Data will be made available for analyses as approved by the data access committee.

Field-specific reporting

Please select the one below that is the best fit for your research. If you are not sure, read the appropriate sections before making your selection.

☒ Life sciences ☐ Behavioural & social sciences ☐ Ecological, evolutionary & environmental sciences

For a reference copy of the document with all sections, see nature.com/documents/nr-reporting-summary-flat.pdf

Life sciences study design

All studies must disclose on these points even when the disclosure is negative.

Sample size	For the current analyses, we used a sub-sample of 736 participants from the RODAM study with DNA methylation (DNAm) data. This epigenetics sub-sample was originally designed to detect 5% DNAm differences between participants with diabetes (~300 diabetic cases,) compared to controls without diabetes (~ 436 obese and non-obese controls), who were equally distributed between migrant and non-migrant groups (case-control design; power=0.80, α=0.05).
Data exclusions	From the 736 participants with DNAm data that were available for this study, 593 participants remained after quality control and excluding participants with possible acute infections (fever, upper respiratory tract symptoms, wound care, any illness in the last 2 weeks), and those taking medications, which may alter CRP levels (immune-modulating agents, NSAIDS, steroids and statins). While CRP concentrations between 2 and 10 mg/L are considered as metabolic inflammation (i.e. metabolic pathways that cause arteriosclerosis),chronically elevated CRP concentrations >10 mg/L have also been associated with CVD. This was particularly important in our study because chronic infections that have higher prevalence in Africans than in populations from HIC (e.g. HIV and Hepatitis C) are also known to raise CRP concentration to >10 mg/L. As such, we sought to include participants with CRP concentrations > 10 mg/L in our analyses while limiting these concentrations to 40 mg/L as CRP levels above this threshold are most likely to be from acute bacterial infections in low and middle income countries (LMIC). Additional removal of participants with CRP levels > 40 mg/L (which were also conspicuous outliers in the CRP distribution) led to a final sample of 589 used in the current analyses.
Replication	We referred to the previous epigenome wide association study (EWAS) meta-analysis on CRP and replicated the reported DMPs in our study. This was achieved by performing an independent statistical analysis on these previously reported DMPs, employing linear regression models like our main DMP analyses. We assumed statistical significance at a nominal p-value of 0.05 (two-tailed).
Randomization	The original 736 participants were selected for DNA profiling based on a case-control design (~300 diabetic cases, ~ 300 controls, ~135 obese controls). The covariates age, sex, alcohol consumption, smoking, estimated cell types and technical effects (hybridization batch and array position) and surrogate variables were included as covariates in all linear regression models. We also included BMI and T2D as covariates in these linear regression models to account for previous RODAM reports, which showed an enrichment for obesity and T2D.
Blinding	Blinding was not relevant in our case as the RODAM study employs a cross-sectional design testing whether DNAm variations are associated with cardiometabolic phenotypes including CRP which was studied in current analyses.

Reporting for specific materials, systems and methods

We require information from authors about some types of materials, experimental systems and methods used in many studies. Here, indicate whether each material, system or method listed is relevant to your study. If you are not sure if a list item applies to your research, read the appropriate section before selecting a response.

Materials & experimental systems

n/a	Involved in the study	
☒	☐	Antibodies
☒	☐	Eukaryotic cell lines
☒	☐	Palaeontology and archaeology
☒	☐	Animals and other organisms
☐	☒	Human research participants
☒	☐	Clinical data
☒	☐	Dual use research of concern

Methods

n/a	Involved in the study	
☒	☐	ChIP-seq
☒	☐	Flow cytometry
☒	☐	MRI-based neuroimaging

Human research participants

Policy information about studies involving human research participants

Population characteristics	The RODAM study enrolled 6385 migrant Ghanaian men and women residing in Europe and Ghana. In Europe, participants were recruited from the cities of Amsterdam (NL), Berlin (DE) and London (UK). In Ghana, recruitment of participants in the urban area was conducted in two purposively chosen cities (Kumasi and Obuasi), while recruitment in the rural area was conducted in 15 villages in the Ashanti region. Participants were included in the present analyses if they were aged ≥ 25

years, had completed the questionnaire, were physically examined, and had blood samples taken.

Recruitment

Engagement of Ghanaian community
The RODAM project, therefore, involved the Ghanaian community leaders in all sites. This included working with religious communities (e.g., churches and mosques), endorsement from local key figures and establishing relationships with healthcare organizations that served these groups. In addition, the project team provided information about the study via local media aimed at the Ghanaian population (e.g., Ghanaian radio and TV stations). Owing to differences in population registration systems across European countries as well as in Ghana, different approaches were needed for the recruitment of the study populations across different locations.

Recruitment strategy in Ghana
In Ghana, two cities (Kumasi and Obuasi) and 15 villages in the Ashanti region served as the urban and rural recruitment sites. The initial sampling frame was the list of enumeration areas (EAs) in the Ashanti region from the 2010 census. A multistage random sampling procedure was adopted to arrive at the sampling of 30 EAs. EAs were stratified, weighted and a random sample of rural and urban EAs was selected. There are over 2000 urban EAs and more than 1000 rural EAs. The first stage was to group the districts into two main categories: districts with a high number of urban (Kumasi and Obuasi) areas and districts with a high number of rural EAs. The next stage of sampling was to put the EAs together in each of the categories and take a weighted random sample of 10 for Kumasi and 5 for Obuasi, respectively. The procedure was repeated for the rural EAs by adding all the EAs in the selected districts and weighted from the first stage together after which a simple random sample procedure was adopted to select the total number of rural EAs (15) required for the study. Letters were sent to all selected health and community authorities to notify them of the start of the study. We sent team members to the various communities to stay among them. Once within the community, the team then organized mini clinics in the field for a period of 1–2 weeks depending on the sampled population and responsiveness of respondents.

Recruitment strategy in the Netherlands
In the Netherlands, Ghanaian participants were randomly drawn from Amsterdam Municipal Health register. This register contains data on country of birth of citizens and their parents, thus allowing for sampling based on the Dutch standard indicator for ethnic origin. All selected participants aged ≥25 years were sent a written invitation combined with written information regarding the study and a opting out response card. Participants were reminded by phone or by home visit after 2 weeks if there is no response. After a positive response, an appointment for physical examination at a local health center was made over the phone followed by a confirmation letter of the appointment, and a digital or paper version of the questionnaire (depending on the preference of the participant) that was sent to the participant's home address.

Recruitment strategy in the United Kingdom
The UK has no population register for migrant groups. Consequently, Ghanaian organizations served as the sampling frame. Lists of these organizations were obtained from the Ghanaian Embassy and the Association of Ghanaian Churches in the UK in the boroughs known to have the greatest concentration of Ghanaians. Lists of all members of their organizations, if available, were also requested, from which several all participants aged ≥25 years were invited to participate in the study. The selected participants were sent a written invitation combined with written information regarding the study and an opting out response card. Participants were sent a confirmation letter of the appointment for a physical examination at a local health center, church, or community center, including a digital or paper version of the questionnaire (depending on the preference of the participant) if they agreed to participate in the study.

Recruitment strategy in Germany
In Berlin, a list of Ghanaian individuals (born in Ghana, or Ghanaian passport holders) was provided by the registration office and was supplemented with contact details of members of Ghanaian organizations and churches in Berlin. From this combined list, all participants aged ≥25 years were invited to participate in the study. In addition, a written invitation combined with written information regarding the study and a response card were sent to the selected participants. Participants were reminded after 2 weeks if there is no response. After a positive response, the participants were contacted by phone to schedule date and location of the interview with a trained research assistant or opt for the digital online version. After the completion of the questionnaire, a date for physical examination was then scheduled.

Ethics oversight

Ethical approval was obtained from ethics committees of involved institutions in Ghana (Kwame Nkrumah University of Science & Technology: CHRPE/AP/200/12), Netherlands (Amsterdam University Medical Center: W12-062#12.17.0086), Germany (Charité University Berlin: EA1/307/12) and UK (London School of Hygiene & Tropical Medicine: 6208) before the start of data collection.17 All participants gave written informed consent.

Note that full information on the approval of the study protocol must also be provided in the manuscript.
